# Characterising the Burden of Work-Related Injuries in South Australia: A 15-Year Data Analysis

**DOI:** 10.3390/ijerph17062015

**Published:** 2020-03-18

**Authors:** Jianjun Xiang, Murthy Mittinty, Michael Xiaoliang Tong, Dino Pisaniello, Peng Bi

**Affiliations:** 1 School of Public Health, The University of Adelaide, Adelaide, SA 5005, Australia; jianjun.xiang@adelaide.edu.au (J.X.); murthy.mittinty@adelaide.edu.au (M.M.); michael.tong@adelaide.edu.au (M.X.T.); dino.pisaniello@adelaide.edu.au (D.P.); 2 School of Public Health, Fujian Medical University, Fuzhou 350108, China

**Keywords:** workers’ compensation, injury claim, occupational injury, South Australia

## Abstract

To characterise the burden of work-related injuries in South Australia, workers’ compensation claim data were obtained from SafeWork South Australia between 2000 and 2014. Descriptive analyses were performed to investigate the burden of work-related injuries by age, gender, occupation, industry, and nature and mechanism of injury. Dunn’s test was used to compare the injury costs and working days lost by industry and occupation. Ordinary linear regression was used to investigate the age-injury cost association. A total of 464,139 workers’ compensation claims were reported during the 15-year period in South Australia, with an overall rate of 4.6 claims per 100 employees, resulting in a total of 20,861,001 working days lost and AU$14.9 billion dollars of compensation payment. Between 2000 to 2014, the annual claim rates, compensation payments, working days lost, and number of work-related death reduced by 59.3, 73.8, 87.1, and 78.6 percent, respectively, while the median compensation payment increased by 67.3% from AU$968 to AU$1620. A 1-year increase in age was associated with a 2.1% (Rate Ratio, RR = 1.021, 95% CI: 1.020–1.022) increase in compensation costs and a 1.3% (RR = 1.013, 95% CI: 1.012–1.020) increase in working days lost. Work-related injury rates are declining in most sectors, however some workers, especially young male technicians and labourers in the community services industry, remain at higher risk. Challenges for workers’ health and safety include the aging labour force, vehicle incidents, and severe injuries among new and foreign-born workers.

## 1. Introduction

Occupational diseases and injuries are a major public health issue, contributing to about 2–14% of the global burden of disease [1]. These not only cause suffering and hardship for the affected workers and their families, but also exert substantial pressure on employers and the broader community through lost productivity and increased use of already overloaded healthcare services [2]. According to Safe Work Australia, an official Australian government organisation in charge of workplace occupational health and safety, the total direct economic cost due to occupational disease and injury was estimated to be AU$61.8 billion for the 2012–2013 financial year, representing 4.1% of Australian GDP (Gross Domestic Product) [3]. Although the burden was shared by employers, workers, and the community, the majority (74%) of the cost was borne by injured workers [3]. Moreover, the proportion of costs borne by workers showed an increasing trend from 44% in 2000 to 77% in 2013 in Australia [3]. 

Analysis of occupational surveillance data can monitor workers’ health and safety, evaluate the effectiveness of workplace interventions, and inform policy development. Workers’ compensation claims are widely used as a proxy for monitoring occupational injuries and accidents in Australia, however despite this, their value has been challenged in recent years [4]. Safe Work Australia provides basic workers’ compensation statistics reports annually [5], however these lack inferential analyses. Moreover, interpretations based on the latest research evidence are not provided in those reports. A further exploratory analysis of these data can be useful to better understand the characteristics of occupational illnesses/injuries and their costs and severity. The aim of this study was to characterize the burden of work-related injuries in South Australia (SA) and to inform ongoing priority identification and policy making.

## 2. Materials and Methods 

Workers’ compensation is a compulsory statutory form of insurance for all employers in Australia [6], covering the vast majority (94.4%) of Australian workers regardless of the type of employment contract in the event of suffering a work-related injury or disease [7]. Although each state has their own workers’ compensation scheme, benefits provided by the compensation insurance systems typically include medical expenses, income maintenance for the period of time an injured worker is off work, or a lump sum payment for a permanent impairment or death [8]. The process of making a compensation claim is basically consistent between jurisdictions [9]. An injured worker who is intending to make a compensation claim must provide his/her employer with injury details in a “claim form”, which should be accompanied by a medical certificate from a general practitioner or other qualified medical practitioner. The employer must then notify the claims management organisation of the claim within a specified time and the claims management organisation has a period of time to determine whether the claim is eligible for workers’ compensation benefits under the legislation and to accept or deny the claim. The Type of Occurrence Classification System (TOOCS) has been developed as coding guidelines for the recording of details of workers’ compensation claims in the following four aspects: the nature of injury, bodily location, mechanism, and agency of injury/disease [10].

All eligible compensation cases are required to be aggregated to each state/territory’s workers health and safety (WHS) regulator (SafeWork SA in South Australia). In South Australia, two private sector insurers operate as scheme agents to manage claims on behalf of the government authority. SafeWork SA maintains a claims database that records the details of each compensation claim. 

Accepted workers’ compensation claims data for South Australia for the period of 1st January 2000 to 31st December 2014 were obtained from SafeWork SA. Variables in the dataset included demographic and employment information (e.g., age, gender, occupation, and industry), injury details (where and when the injury occurred and injury descriptions), and outcome information (e.g., time lost from work and compensation expenditure). Time lost from work refers to the total number of days a worker was absent from work due to a work-related injury. Compensation expenditure is the lump sum payment to an injured worker to close off a claim, comprising of income maintenance and medical expenses. Serious claims were defined as those resulting in more than two weeks of compensated time loss [7]. The study was approved by the Human Research Ethics Committee at the University of Adelaide (H-111-2011) and the SafeWork SA data custodian.

Descriptive analyses were performed to summarize the characteristics of injury claims and costs by age, gender, occupation (Australian Standard Classification of Occupation, ASCO), industry (South Australia WorkCover Industrial Classification, SAWIC), and the nature and mechanism of injury claims. Percentiles were used for highly skewed variables such as days lost from work and compensation costs. Interquartile range (IQR = Q uartile3-Quartile1) was calculated measuring working days lost. The unit of currency for measuring compensation costs was the Australian dollar (AU$). Compensation claims registered in the metropolitan area of the city Adelaide (capital of the state of South Australia) were identified by injury location postcode (5000–5199 and 5900–5999). The direct age-standardisation method was used for calculating injury claim rates [11], with age group intervals of 15–24, 25–34, 35–44, 45–54, 55–64, and 65+ years. The denominators (insured labour force estimates by age, gender, industry, and occupation for 2000–2014) were obtained from Safe Work Australia (a national agency responsible for WHS and workers’ compensation arrangements), which were jointly developed by Safe Work Australia and Australian Bureau of Statistics (ABS) after taking partial coverage of current compensation insurance into account. The standard labour force used for calculating age-standardised rates was the investigated labour force in the 2006 Australian Population Census [12]. Claim rates and death rates were used to reflect the frequency of work-related morbidity and mortality, respectively. 

Case fatality rate was calculated to reflect the fatal risk of an injury, as shown in Equation (1) below:

(1) $$\mathrm{Case}\;\mathrm{fatality}\;\mathrm{rate}=\frac{\mathrm{Number}\;\mathrm{of}\;\mathrm{Deaths}\;\mathrm{from}\;\mathrm{an}\;\mathrm{Injury}}{\mathrm{Number}\;\mathrm{of}\;\mathrm{workers}\;\mathrm{with}\;\mathrm{that}\;\mathrm{injury}}$$

Given the possible impact of currency inflation overtime, the variable “expenditure” was adjusted by multiplying the ratio of quarterly health services related consumer price index (CPIs) in 2014 by each CPI for the years of 2000 to 2013. CPI data were downloaded from the Australian Bureau of Statistics website [13].

All analyses were performed with Stata V.15.1 (StataCorp LP, College Station, Texas, USA). Normality of the data was checked using Shapiro-Wilk tests and histograms. Dunn’s test [14] was used for pairwise multiple comparisons of compensation costs and working days lost by industry, occupation, nature of injury, injury mechanism, new worker (yes/no) (the duration of work less than one year or those aged ≤18 years), business size, and country of birth. Rate differences between males and females were checked using Pearson’s chi-square tests. Ordinary linear regression was used to quantify the associations between age and log-transformed compensation expenditure and working days lost (to account for skewed data), after adjusting for gender, occupation, industry, nature of injury, and the impact of early reporting incentives (ERIs) by creating a dummy variable in the model. ERIs were introduced as a policy in 2009 in South Australia, encouraging employers to report worker injuries in a timely fashion. Employers who lodged a compensation claim within two working days of becoming aware of an injury were given a rebate on their insurance excess [15]. Results for the regression models were expressed as rate ratios (RR) with 95% confidence intervals (CIs) by exponentiating the coefficients and interpreting them as a percentage change in compensation expenditure or working days lost with change of age. The association between age at injury and injury severity was examined using logistic regression after controlling for gender, industry, occupation, and nature of injury. The 0.05 (*p*-value) level of statistical significance for two tails was adopted for each test.

## 3. Results

**Figure 1:**
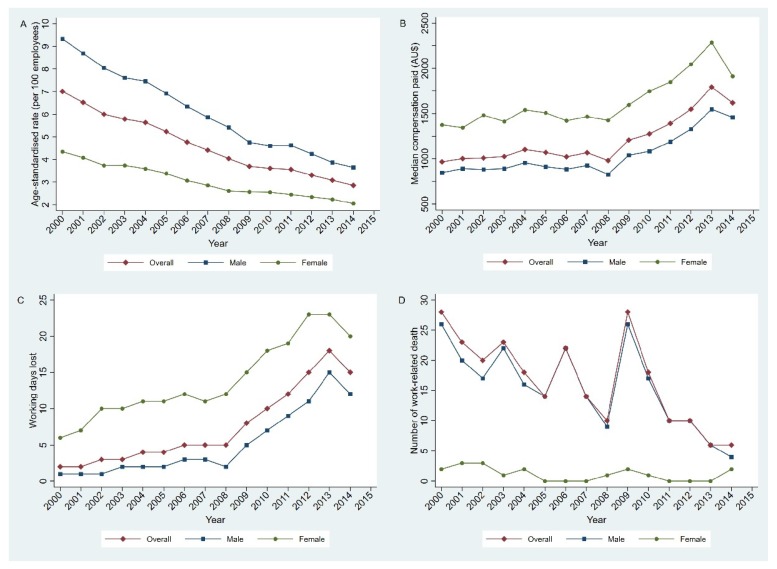
Trends in workers’ compensation claim statistics in South Australia, 2000–2014: (**A**) age-standardised claim rates, (**B**) median compensation payments, (**C**) interquartile range of working days lost, and (**D**) the number of work-related deaths.

There were 464,139 notified workers’ compensation claims during the 15-year period between 2000 and 2014 in South Australia. There was an overall claim rate of 4.6 per 100 employees, resulting in a total time lost of 20,861,003 working days in 2000–2014 and AU$14.9 billion dollars in compensation payments. A gradual downward trend was observed over time for claim counts, claim rates, time lost, and compensation expenditure. The number of claims more than halved from 42,002 in 2000 to 19,406 in 2014. About 20% were serious claims (resulting in more than two weeks’ time loss). The claim rate reduced by 59.3% from 7.0% in 2000 to 2.9% in 2014 (Figure 1A). Between 2000–2014, time lost and injury claim costs reduced 87.1% from 1,813,717 days to 474,619 days and 73.8% from AU$1.7 billion to AU$0.2 billion, respectively. However, median costs for overall compensation claims increased by 67.3% from AU$968 in 2000 to AU$1,620 in 2014 (Figure 1B). For serious claims, however, median compensation payments reduced by 54% in 2000–2014 (Supplementary Figure S1B). The interquartile range for non-serious claims increased since 2008 (Supplementary Figure S2C), while for serious claims, it reduced by 56.8% overtime (Supplementary Figure S1C). A total of 250 workers died from work-related injuries in South Australia over the study period, with an overall mortality rate of 2.5 per 100,000 employees. The number of deaths reduced by 78.6% from 28 in 2000 to 6 in 2014, as shown in Figure 1D.

### 3.1. Age and Gender

**Figure 2:**
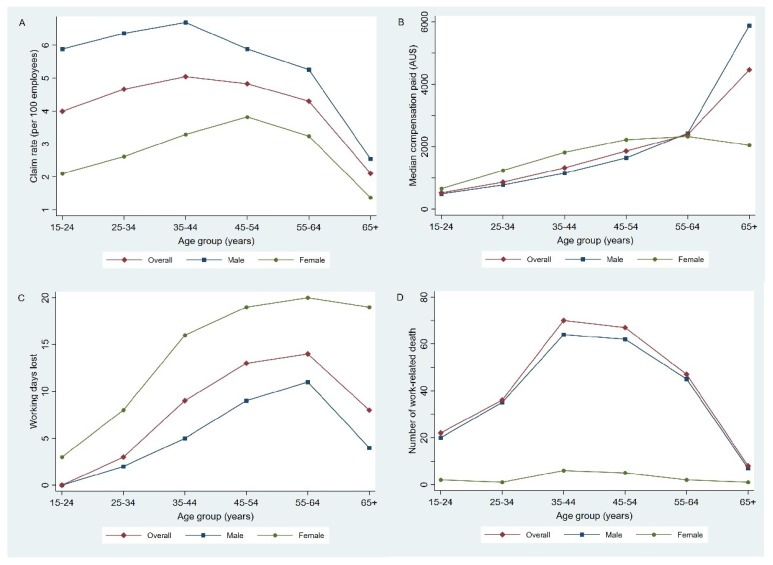
Workers’ compensation claims for 2000–2014 by age group in South Australia showing: (**A**) Age-standardised claim rate, (**B**) median compensation payment, (**C**) interquartile range of working days lost, and (**D**) number of work-related deaths.

The number of injury claims lodged by male workers (316,513) was more than two times that of female workers (147,599) over the study period. Moreover, the claim rate for male workers (6.0 per 100 employees) was significantly higher than that of female workers (3.0 per 100 employees) (*p* < 0.05, Supplementary Table S1). As shown in Figure 2A, claim rates increased with the increase of age for both male and female claimants until 35–44 years and then dropped to the lowest level at the 65+ age group. The median compensation expenditure increased with age for both genders (Figure 2B). The median compensation cost of claims for females was significantly higher than that for male claimants in the 15–64 years group (χ^2^ = 2592.391, *p* < 0.001), while it was the opposite for the 65+ age group (χ^2^ = 45.937, *p* < 0.001). Similarly, the interquartile range of working days lost increased with age until 55–64 years for both genders and then dropped slightly for those aged 65 and over, as shown in Figure 2C. Moreover, female claimants had consistently more median working days lost than male claimants in all age groups. 

Results of ordinary linear regression showed that a 1-year increase in age was associated with a 2.1% (RR = 1.021, 95% CI: 1.020–1.022) increase in compensation expenditure and a 1.3% (RR = 1.013, 95% CI: 1.012–1.020) increase in working days lost. For serious claims, a 1-year increase in age was associated with a 1.5% (RR = 1.015, 95%CI: 1.014–1.017) and 0.8% (RR = 1.008, 95%CI: 1.007–1.010) increase of compensation payments and working days lost, respectively. The proportion of serious claims increased with the increase of age group (Supplementary Table S2). More than half (54.8%) of work-related deaths occurred in the 35–54 age group (Figure 2D), while the highest death rate was observed in the 55–64 age group at 3.1 per 100,000 employees (Supplementary Table S1). The vast majority (93.2%) of work-related deaths were males.

### 3.2. Industry

As shown in Table 1, approximately two-thirds of work-related injury claims occurred in three industries: “community services” (28.2%), “manufacturing” (26.0%), and “wholesale and retail trade” (15.9%). The manufacturing industry had the highest injury claim rate (10.1 per 100 employees), followed by “transport and storage” and “construction”. Results of age, gender, and industry stratified analyses showed that the claim rate of male community service workers aged 15–24 years reached as high as 22.4 per 100 employees (Supplementary Figure S3). Specifically, they mainly were “structural construction, automotive, fabrication engineering, foods, electrical, and electronics tradespersons” (30.6%) and “process workers” (12.1%). More than two-thirds of claims from this subgroup were caused by wounds and traumatic injuries. For female workers, the highest rate was observed among those aged ≥65 years in the “electricity, gas, and water” industry at 18.2 per 100 employees, however there were only two injury claims. Male workers almost had higher claim rates than female workers in all age groups and industries. 

Results of pairwise multiple comparisons showed that “public administration and defence” (AU$ 1456) had significantly higher median compensation payments for non-serious claims than other industries, while for serious claims, significantly higher median compensation payments were found in “communication’ (AU$ 65,603), “mining” (AU$ 50,171), “finance, property, and business services” (AU$ 49,566), and “manufacturing” (AU$ 46,925). Relatively more working days lost per claim (IQR) for serious claims were found in “communication” (107 days) and “finance, property, and business services” (87 days), compared with other industries (Table 1). Usually, female claimants aged less than 65 years had higher median compensation expenditure than their male counterparts (Supplementary Figures S4 and S5). The highest median compensation costs (AU$ 552,391) due to serious claims were observed in male claimants aged 35–44 years in the communication industry (Supplementary Figure S5). Of them, 28.5% were labourers (e.g., process workers) and 28.5% were miscellaneous tradespersons. Generally, female claimants had more working days lost due to serious injuries than that from male claimants, excluding those employed in construction and the communication industry (Supplementary Figure S6). 

In terms of work-related deaths, about one-quarter (64) occurred in the “transport and storage” industry, with the highest rate of 16.1 per 100,000 employees compared with other high risk industries such as “construction” (7.5 per 100,000 employees) (Table 1). The majority (71.9%) of work-related deaths in the “transport and storage” industry resulted from vehicle incidents.

### 3.3. Occupation

As shown in Table 2, the top three occupations with the highest percentage of total claims were “labourers” (27.0%), “technicians and trades workers” (23.2%), and “machinery operators and drivers” (16.3%), respectively, accounting for about two-thirds of total claims. Machinery operators and drivers had the highest compensation claim rate (11.2 per 100 employees), followed by labourers (9.9 per 100 employees) and technicians (8.3 per 100 employees). Usually, female workers had a lower claim rate than male workers, while it was the opposite for female professionals (all age groups), female managers and sales workers (all age groups), and young female machinery operators (15–34 years) when compared with their male counterparts (Supplementary Figure S7). 

For serious claims, relatively higher median compensation payments were observed in “managers” (AU$ 53,954), “professionals” (AU$ 45,466), and “clerical and administrative workers” (AU$ 44,460) (Table 2). Except for community and personal services related workers, female claimants aged ≤64 years usually had higher compensation claims expenditures than their male counterparts for both serious and non-serious claims (Supplementary Figures S8 and S9). However, when age exceeded 65 years, it is the opposite in most industries. 

Regarding working days lost due to serious injury claims, the three occupations (managers, sales workers, and clerical and administrative workers) had relatively more work time lost (interquartile range) than other occupations (Table 2). Generally, female claimants, regardless of age group and occupation, had consistently more working days lost than male claimants due to serious injuries (Supplementary Figure S10). Machinery operators and drivers not only had the highest percentage (38.4%) of work-related deaths, but also the highest death rate (11.8 per 100,000) (Table 2). Vehicle incident was the leading cause (65.6%) of death for machinery operators and drivers.

### 3.4. Nature of Injury

As shown in Table 3, the leading causes of work-related injuries were “traumatic joint/ligament and muscle/tendon injuries” (43.2%), “wounds, lacerations, amputations, and internal organ damage” (22.1%), and “musculoskeletal and connective tissue diseases” (11.6%). They accounted for 71.3% of total compensation expenditure and 72.1% of total working days lost. For non-serious claims, female claimants had higher median compensation payments than male claimants due to “musculoskeletal diseases” and “infectious and parasitic diseases” in almost all age groups (Supplementary Figure S11). While for serious claims, male claimants constantly had higher median compensation payments than female claimants in almost all age groups for all conditions (Supplementary Figure S12). Moreover, noticeably high median compensation payments for serious claims were observed in the following conditions: neoplasms in male claimants aged 15–24 years, circulatory diseases in male claimants, and injury to nerves and spinal cord in the 45–54 years age group. Serious conditions such as circulatory diseases, injury to nerves and spinal cord, and intracranial injuries usually caused more working days lost (Supplementary Figure S13). 

Among the 250 work-related deaths, 16.8% were classified as circulatory diseases, followed by “wounds, lacerations, amputations, and internal organ damage” (8.0%) and intracranial injuries (6.0%). In terms of case fatality rate, circulatory diseases (7554 per 100,000 claims) were remarkably higher than other diagnosis categories (Table 3).

### 3.5. Mechanism of Injury

As shown in Table 4, 38.5% of injury claims were attributable to body stressing, which disproportionately accounted for 48.6% of total compensation expenditure and 49.5% of working days lost. Mental stress and vehicle incidents occupied 3.1% and 3.9% of total claims, while the percentages for compensation payments were 11.3% and 7.1%, respectively. Disproportionately high percentages were also observed in working days lost. Mental stress (AU$ 73,111) had the highest median compensation payment due to serious claims, while for minor claims, it was “sound and pressure” (AU$ 25,589). Generally, male claimants had higher median expenditures than female claimants in all age groups and all injury mechanisms for serious claims (Supplementary Figure S15). Serious claims due to mental stress, vehicle incidents, sound and pressure in male claimants aged 55–64 years and chemical exposure in male claimants aged 65+ years caused relatively more compensation payments. Correspondingly, they resulted in relatively more working days lost (Supplementary Figure S16).

“Vehicle incident” was the most common type of injury mechanism (48.8%) resulting in work-related death. In addition, vehicle incidents had the highest fatality rate (671 per 100,000 claims). More than three-quarters (78.0%) of vehicle incident-related claims occurred in the Adelaide metropolitan area.

### 3.6. Work Experience, Business Size, and Birth Place

As shown in Table 5, claims lodged by new workers accounted for 15.6% of total claims. New workers had less median compensation payments but significantly more working days lost due to serious claims than that of non-new workers (χ^2^ = 144.5, *p* < 0.001). Although the injury fatality rate of new workers was slightly higher than non-new workers (69 vs. 51 per 100,000 claims), no statistical difference was detected (χ^2^ = 3.688, *p* = 0.055). 

Large businesses accounted for more than half (51.9%) of injury claims, while they had the lowest percentage of work-related deaths (21.6%). Results of Dunn’s test showed that median injury compensation expenditure was negatively associated with business size. Large and medium businesses had significantly higher median compensation costs than small businesses. However, serious claims from small and medium businesses caused significantly more working days lost than that of large businesses. Small businesses recorded 16.6% of total claims, however disproportionately accounted for 42.8% of work-related deaths. Moreover, small businesses had a significantly higher case fatality rate than large and medium businesses (χ^2^ = 150.917, *p* < 0.001). 

The majority of injury claimants were born in Australia (73.4%), were native English speakers (97.3%), and had significantly lower median compensation claims expenditure (χ^2^ = 298, *p* < 0.001) and working days lost (χ^2^ = 227, *p* < 0.001) than those born overseas and non-native English speakers, respectively. As shown in Supplementary Table S3, except for Central-South Asia and Pacific Island countries, foreign-born claimants had significantly higher median compensation cost and working days lost than Australia-born claimants, especially those born in Middle-East countries. About two-thirds of these Middle-East born claimants were males and one-third of them were labourers. Traumatic joint/ligament and muscle/tendon injury (48.9%) was the major cause for them to make claims. The highest case fatality rate was observed in claimants born in North-East Asia (148 per 100,000 claims), followed by Pacific Island countries (109 per 100,000 claims), indicating that these overseas workers might undertake the higher risk jobs.

## 4. Discussion

Learning from past occupational injuries/accidents is an important component for improving workers’ health and safety, preventing the recurrence of similar injuries, identifying high-risk groups, and providing empirical evidence for decision making and resources allocation. Evidence has shown that many work-related injuries/illnesses are preventable through effective engineering and ergonomics controls, introducing new regulations, conducting inspections, industry campaigns, and WHS training [16,17,18]. Moreover, workplace interventions for health and safety have been proven to have long-term economic benefits for employers and the whole of society [19], making the incremental investment in occupational health and safety worthwhile. The Australian Work Health and Safety Strategy 2012–2022 was established by Safe Work Australia to improve workplace health and safety and reduce work-related morbidity and mortality by at least 30% and 20% by 2022, respectively [20]. In this study, the total direct compensation expenditure during 2000–2014 was roughly equivalent to 15.0% of the state’s GDP in 2014. A better understanding of the magnitude and burden of different occupational hazards can provide essential information for targeted risk reduction and facilitate the fulfilment of goals.

### 4.1. A Downward Trend: Implications for Intervention Effectiveness Evaluation

This study depicted an overall picture of the epidemiological characteristics of workers’ injury claims and relevant economic burden in South Australia for the period 2000–2014. Consistent with the national report [5] and in accordance with the aims of the Australian Work Health and Safety Strategy, both work-related morbidity and mortality are demonstrating a gradual downward trend. This may be partly due to continuous occupational health and safety efforts and improvements of working conditions. Other possibly important contributing factors could be (1) the shift from a manufacturing and industrial economy to a service-based one. According to our data analysis, the number of workers in the manufacturing industry in South Australia reduced about 18% during 2000–2014, meanwhile there has been about a 10% increase in the community services sector during the same period; (2) the shift from manual work to automation [21], which has led to a reduction of workers exposed to hazardous workplace conditions. In the process of deindustrialisation, a gradual decline in work-related morbidity and mortality has also been observed in other developed countries such as USA [22] and Canada [23]; and (3) the tightening eligibility for compensation benefits overtime. Evidence has shown that the level of compensation benefits was positively associated with claim rate [15,24]. In addition, the trend might be confounded by the introduction of ERIs in 2009, although there is limited research to investigate its impact on injury claims. Therefore, the changes in industrial structure and labour force characteristics should also be taken into account when evaluating the effectiveness of mid-and-long term WHS interventions such as legislative changes. 

During the study period, the net cost of injury claims and working days lost reduced by 74% and 87%, respectively. Although median compensation claims payments (adjusted for currency inflation) and working time lost due to minor injuries increased over time, for serious claims they both reduced more than 50%, indicating that Australian workplaces have become safer over the past decade [7].

### 4.2. Identification of Local Specific Priorities for Targeted Risk Reduction

Priority setting depends on a regulator’s goals relating to three domains: reduction of work-related deaths, reduction of injury incidence rate, and reduction of severe injuries/diseases. Although the national priorities have been released by Safe Work Australia, they may be too broad to meet the varying conditions of each state [20]. Therefore, there is a need to identify local specific priorities for targeted interventions. 

Our results suggest that male community service workers aged 15–24 years were at the highest risk of work-related injuries. They accounted for 0.5% of the total labour force in South Australia, however the claims they lodged disproportionately occupied 2.6% of total claims and 1.5% of serious claims. Although the median compensation payments and working days lost from this group of workers are not noticeable compared to other subgroups, interventions on them may be of benefit by reducing the injury incidence rate. We also found machinery operators and drivers, labourers, and technicians were the top three occupations at high risk of injury in the workplace. Joint/ligament and muscular trauma was the most common work-related injury and body stressing was the most common cause of work-related injuries. These findings may inform the development of interventions to reduce injury incidence rates. 

To reduce the occurrence of severe injuries resulting in higher compensation payments and more working days lost, efforts may need to focus on male labourers and tradespersons aged 35–44 years in the communication industry and claims due to circulatory diseases, injury to nerves, intracranial injuries, and mental disorders. Vehicle incidents accounted for about 50% of work-related deaths in the present study. This compares with overseas figures, which show that vehicle incidents account for 22% of work fatalities in the USA and 16% in New Zealand [25]. It highlights the need to focus more efforts on road risk management practices in the workplace, especially for male machinery operators and drivers employed in small businesses in the transport and storage industry [26]. 

Generally, the length of service influences a worker’ skills and attitudes towards occupational safety [27], and workers with shorter length of service are at high risk of various injuries [28]. A literature review by Khanzode suggested that both newer workers and highly experienced workers were at high risk of injuries [29]. In this study, we found that new workers (less than 1 year with an employer) had lower median compensation payments but higher working time lost compared to non-new workers. This implies that new workers had a relatively lower salary and were at higher risk of severe injuries than non-new workers in the workplace due to peer pressure and their inexperience. 

Consistent with previous literature, we found that workers in small businesses were more likely to sustain severe and fatal injuries due to the unavailability of quality occupational health services and facilities [30], compared with large-medium businesses. A recent comprehensive literature review suggested that different intervention methods worked differently for large and small businesses [17]. Intervention approaches developed specifically for large businesses may not be automatically transferred to small businesses directly [31]. Regulators may need to consider providing tailored advice according to business size, rather than a one size fits all approach. For small businesses in particular, it may be more effective to provide easy access to information and support to enable them to become compliant [17].

Australia is a nation of migrants and one in three Australians are born overseas [32]. In this study, we found foreign-born workers, especially those from Middle-East countries, had relatively more compensation payments and more working days lost per claim than that of Australia-born workers. Moreover, injury claimants from North-East Asia and Pacific island countries had significantly higher case fatality rates than other claimants. This implies that foreign-born workers are at higher risk of severe injuries than Australia-born workers, assuming no differences in the reporting of minor injuries. Similar findings have been reported in studies from Canada and the USA. Smith et al. found that the risk of serious work-related injuries requiring medical treatment in foreign-born workers was about two times higher than Canadian-born workers in 2003–2005 [33]. Similarly, Zhang et al. found that compared to US-born workers, the injuries of foreign-born workers were more likely to result in hospitalisation and a greater proportion of injuries requiring ≥ 6 days off work in the USA during 1997–2005 [34].

### 4.3. Gender Differences

Our results showed that male workers’ claim rates were higher than that of female workers in blue-collar occupations, especially in some labour-intensive industries such as manufacturing, mining, and construction. In addition, work-related death rates of male workers were significantly higher than female workers in all age groups. This is in line with overseas findings showing that men had a three times higher injury rate than women in Finland and Canada [35]. This may be due to the over-representation of males in high-risk industries. Moreover, male workers are more likely to undertake high-risk tasks in the workplace. Another potential reason resulting in gender differences in claim rates may be that female workers were less likely than male workers to apply for workers’ compensation for their work-related injuries [36]. According to the Work-Related Injuries Survey (WRIS) conducted by the ABS for the period of 2005–2006 [36], 44% of female workers who sustained injuries that resulted in some time lost from work did not apply for a compensation claim, compared to 36% for male workers. 

Our results also showed that claim rates of male workers decreased as age increased in most industries. Higher non-fatal injury rates among young male workers have also been observed in other countries (e.g., USA) [37,38], although young workers can be less likely to make injury claims than older workers according to the ABS WRIS survey [36]. Young male workers’ vulnerability to injury in the workplace has generally been attributed to lack of occupational risk awareness, training, and relevant work skills, together with peer pressure [38]. Young workers’ health and safety have been listed as one of the occupational research priorities by Safe Work Australia [20]. 

Although female claimants aged 15–54 years in almost all industries had lower claim rates than their male counterparts, they usually had higher compensation payments and took longer to return to work. One possible explanation is that female workers aged 15–54 years may have filed a smaller percentage of claims related to minor injuries and selectively reported the severe injuries only [36]. For the age group of 65+ years, male claimants had higher claim rates and more compensation payments but lower working days lost per claim compared to female claimants. The inconsistency between compensation payment and working time lost may be due to the high gender payment gap in the 65+ year age group. Statistics from the Australian Workplace Gender Equality Agency showed that the average gender pay gap increased with age, reaching 17% in the age group of 55+ years [39]. Therefore, working time lost is a better indicator to reflect injury severity than compensation payment as workers’ compensation insurance not only covers an injured worker’s medical treatment costs, but also a portion of the employee’s wage and a lump sum payment when the employee suffers a permanent impairment [6]. Evidence has shown that female workers aged over 65 years had a lower underreporting rate of minor injuries than male workers in the same age group [36]. Therefore, more working days lost per claims among older female workers implies that they are truly (not biased by underreporting of minor injuries) at higher risk of non-fatal serious injuries than male workers in most industries except “agriculture, forestry, fishing, and hunting”, mining, and construction. However, the low rates of males and females working after the retirement age requires cautious interpretation of this result. 

### 4.4. Injury Severity: Growing Challenges of an Aging Labour Force

Our results showed that the proportion of serious claims increased with the increase of age. Moreover, a 1-year increase in age was associated with a 3.7% and 1.3% increase of compensation expenditure and working time lost, respectively, indicating that older workers sustained more severe injuries than their younger counterparts and required more days away from work to recover [35,40,41]. Schwatka et al. attributed this to older workers’ selectively reporting serious injuries due to worries of negative attention for their carelessness or unsafe behaviours [42]. Moreover, higher compensation costs among older workers have been shown to result from indemnity costs rather than medical treatment expenditure [42]. Nevertheless, we found that older workers had a higher work-related death rate than young workers, which is consistent with previous studies [35]. This could be because young workers are physically healthier [43].

The aging labour force, due in part to the raising of the retirement age in recent years, has led to increased numbers of older workers in the workforce [44]. According to ABS data [12,45], the percentage of workers aged 65+ has increased about 2.5 times from 1.6% in 2000 to 3.9% in 2014 in South Australia. This may present a growing challenge for the reduction of severe injuries and related compensation payments and the fulfilment of the Australian Work Health and Safety Strategy 2012–2022, which aims to reduce severe claims by at least 30% by 2022 [20]. Potentially increasing severe injuries among older workers would not only affect the well-being of the workers and their families, but also financially burden the employers, insurance companies, and our society. 

### 4.5. Limitations

There are several limitations to this study. First, workers’ compensation claims may fail to capture all work-related illnesses and injuries due to under-reporting. Claiming access to compensation is not merely a result of a biomedical injury. A range of policy, demographic, societal, and psychological factors affects the reporting of injuries and the decision to lodge a compensation claim [7]. According to the Australian Bureau of Statistics Work-related Injury Survey, only 35.9% of workers who experienced work-related injuries received compensation payments in 2010, while the percentage increased to 80% for serious injuries causing more than one-week time loss [46]. This means that the discrepancy for serious injuries tends to be small. Second, compensated time loss recorded in the data generally underestimates the amount of time an injured worker is away from work [9]. Third, the amount of compensation payment might be affected by the change in compensation policies. For example, in 2000–2008, weekly payments for income maintenance were equal to the worker’s national weekly earnings for the period of incapacity for work not exceeding one year [47]. Afterward, only the first 13 weeks are entitled to weekly payments equal to the national weekly earnings [6]. Therefore, the tightening compensation benefits may be partially contributing to the reduced compensation payments during the study period. Fourth, to capture the overall picture of work-related injuries and its burden, the injury claims included in the analysis were not right-censored to ensure the consistency of follow-up duration between earlier and latter claims in the study period. Nevertheless, we found that the data were fairly stable in 2000–2013. There are some ongoing active claims (immature cases) in 2014. This may partly contribute to the slight drop in median compensation payment and IQR working days lost in 2014, in addition to a major change to the compensation policies in 2014 which tightened the compensation benefits.

## 5. Conclusions

Work-related injuries are declining in most industrial sectors, but some workers remain at higher risk. To reduce injury rates, more attention should be paid to young male technicians and labourers in the community services industry and injuries caused by body stressing. Challenges for workers’ health and safety include the aging labour force, vehicle incidents, and severe injuries among new and foreign-born workers.

## Figures and Tables

**Table 1:** The number of claims, percentage, compensation payment, time-loss days, and deaths by industry, South Australia, 2000–2014.

SAWIC Industrial Classification	Claims: n (%)	Claim Rate (per 100)	Total Cost (Million): n (%)	Median Cost for Serious/Non-Serious Claims	Total Time-Loss Days: n (%)	Time-Loss Days for Serious Claims: Median (25–75th Percentile)	Death: n (%)	Death Rate (per 100,000)
Community Services ^†^	132,361 (28.2)	5.9	4242 (28.4)	34,815/764	6,069,528 (29.1)	59 (27–185)	35 (14.0)	1.5
Manufacturing	120,511 (26.0)	10.1	3479 (23.3)	46,925/662	4,029,229 (19.3)	52 (26–193)	33 (13.2)	2.8
Wholesale and Retail Trade	73,668 (15.9)	4.6	1947 (13.0)	37,262/570	3,025,014 (14.5)	62 (29–252)	21 (8.4)	1.6
Construction	33,157 (7.1)	6.0	1409 (9.4)	37,812/562	2,054,175 (9.8)	65 (30–302)	39 (15.6)	7.5
Recreation, Personal, and Other Services	24,365 (5.3)	2.1	759 (5.1)	29,608/533	1,351,372 (6.5)	63 (28–243)	8 (3.2)	0.8
Transport and Storage	23,774 (5.1)	6.2	1109 (7.4)	41,096/753	1,528,404 (7.3)	64 (29–262)	64 (25.6)	16.1
Finance, Property, and Business Services	17,198 (3.7)	1.2	754 (5.0)	49,566/739	1,251,742 (6.0)	87 (34–375)	15 (6.0)	1.0
Public Administration and Defence	15,549 (3.4)	2.6	258 (1.7)	26,196/1,456	242,829 (1.2)	34 (21–74)	* (*)	0.3
Agriculture, Forestry, Fishing, and Hunting	14,255 (3.1)	4.4	623 (4.2)	26,540/546	978,519 (4.7)	55 (28–198)	22 (8.8)	6.5
Mining	5397 (1.2)	4.5	280 (1.9)	50,171/813	275,133 (1.3)	56 (27–210)	10 (4.0)	7.1
Electricity, Gas, and Water	3612 (0.8)	3.0	49 (0.3)	36,230/751	22,788 (0.1)	35 (22–75)	* (*)	0.8
Communication	257 (0.1)	0.2	21 (0.1)	65,603/724	29,580 (0.1)	107 (45–471)	0 (0.0)	0.0
Non-Classifiable	35 (0.01)	-	3 (0.02)	107,549/2,506	2690 (0.01)	188 (113–373)	0 (0.0)	-
Total	464,139(100.0)	4.6	14,934 (100.0)	37,428/671	20,861,003 (100.0)	58 (27–210)	250 (100.0)	2.5

*: Counts ≤5 were suppressed for privacy protection; SAWIC: South Australian Industry Classification; ^†^: Community services mainly comprise health care and social assistance, education and training, police services, fire brigade services, employment services, and waste disposal services; Discrepancies may occur between sums of the component items and totals due to missing values.

**Table 2:** The number of claims, percentage, compensation payment, time-loss days, and deaths by occupation, South Australia, 2000–2014.

Occupation Classification	Claims: n (%)	Claim Rate (per 100)	Total Cost (Million): n (%)	Median Cost for Serious/Non-Serious Claims	Total Time-Loss Days: n (%)	Time-Loss Days For Serious Claims: Median (25–75th Percentile)	Death: n (%)	Death Rate (per 100,000)
Labourers	125,043 (27.0)	9.9	4110 (27.5)	35,554/626	6,186,878 (29.7)	57 (27–227)	44 (17.6)	3.3
Technicians and Trades Workers	107,766 (23.2)	8.3	2662 (17.8)	33,564/523	3,511,634 (16.8)	50 (26–176)	47 (18.8)	3.0
Machinery Operators and Drivers	75,787 (16.3)	11.2	2829 (18.9)	42,829/753	3,654,779 (17.5)	60 (28–235)	96 (38.4)	11.8
Community and Personal Service Workers	53,504 (11.5)	5.0	1714 (11.5)	29,927/867	2,702,035 (13.0)	56 (27–175)	10 (4.0)	1.0
Professionals	35,172 (7.6)	1.7	1276 (8.5)	45,466/1,045	1,473,488 (7.1)	64 (30–189)	22 (8.8)	1.1
Sales Workers	30,409 (6.6)	3.2	879 (5.9)	40,113/628	1,347,844 (6.5)	65 (30–251)	* (*)	0.5
Clerical and Administrative Workers	21,820 (4.7)	1.2	753 (5.0)	44,460/1,064	1,072,727 (5.1)	66 (29–215)	* (*)	0.3
Managers	14,460 (3.1)	1.3	699 (4.7)	53,954/837	891,038 (4.3)	84 (34–318)	22 (8.8)	1.2
Unknown	178 (0.04)	-	12 (0.1)	82,212/493	20,578 (0.1)	107 (49–643)	* (*)	-

*: Counts ≤ 5 were suppressed for privacy protection. Discrepancies may occur between sums of the component items and totals due to missing values.

**Table 3:** The number of claims, percentage, compensation payment, time-loss days, and deaths by nature of injury, South Australia, 2000–2014.

Nature of Injury	Claims: n (%)	Total Cost (Million): n (%)	Median Cost for Serious/Non-Serious Claims	Total Time-Loss Days: n (%)	Time-Loss Days For Serious Claims: Median (25–75th Percentile)	Death: n (%)	Case Fatality Rate (per 100,000)
Traumatic joint/ligament and muscle/tendon injury	220,456 (43.2)	6280 (42.1)	35,038/944	8,440,125 (40.5)	53 (25–176)	*(*)	1
Wounds, lacerations, amputations, and internal organ damage	102,481 (22.1)	807 (5.4)	21,568/388	976,560 (4.7)	35 (22–81)	20 (8.0)	20
Musculoskeletal and connective tissue diseases	53,983 (11.6)	3551 (23.8)	57,401/1,703	5,615,172 (26.9)	86 (34–378)	* (*)	4
Fractures	19,888 (4.3)	1024 (6.9)	24,702/1,292	1,554,378 (7.5)	50 (29–493)	12 (4.8)	60
Nervous system and sense organ diseases	15,167 (3.3)	608 (4.1)	36,980/4,505	622,725 (3.0)	57 (29–194)	* (*)	0
Mental diseases	14,722 (3.2)	1699 (11.4)	72,936/3,046	2,570,326 (12.3)	132 (51–355)	* (*)	34
Burns	10,434 (2.3)	96 (0.6)	20,182/374	95,887 (0.5)	28 (19–57)	* (*)	48
Skin and subcutaneous tissue diseases	7161 (1.5)	92 (0.6)	17,650/429	131,182 (0.6)	36 (20–107)	* (*)	0
Digestive system diseases	4464 (1.0)	113 (0.8)	17,584/6,995	171,047 (0.8)	31 (22–46)	* (*)	22
Intracranial injuries	3317 (0.7)	130 (0.9)	59,245/627	137,222 (0.7)	74 (29–493)	15 (6.0)	452
Respiratory system diseases	1332 (0.3)	62 (0.4)	45,643/1,764	79,106 (0.4)	73 (36–194)	* (*)	0
Infectious and parasitic diseases	1033 (0.2)	20 (0.1)	16,170/768	27,510 (0.1)	37 (21–102)	* (*)	194
Injury to nerves and spinal cord	557 (0.1)	62 (0.4)	33,456/2,176	63,203 (0.3)	57 (25–441)	* (*)	0
Circulatory system diseases	556 (0.1)	110 (0.7)	106,762/1,667	102,090 (0.5)	99 (46–453)	42 (16.8)	7554
Neoplasms (cancer)	157 (0.03)	10 (0.1)	70,511/5,254	4717 (0.0)	37 (18–348)	* (*)	1911
Other	28,431 (6.1)	146 (1.0)	54,939/293	269,735 (1.3)	76 (30–394)	141 (56.4)	496
Total	464,139 (100.0)	14,934 (100.0)	37,428/671	20,861,003 (100.0)	58 (27–210)	250 (100.0)	54

*: Counts ≤5 were suppressed for privacy protection; Discrepancies may occur between sums of the component items and totals due to missing values.

**Table 4:** The number of claims, percentage, compensation payment, time-loss days, and deaths by mechanism of injury, South Australia, 2000–2014.

Mechanism of injury	Claims: n (%)	Total cost (million): n (%)	Median cost for serious/non-serious claims	Total time-loss days: n (%)	Time-loss days for serious claims: median (25–75th percentile)	Death: n (%)	Case fatality rate (per 100,000)
Body stressing	178,824 (38.5)	7253 (48.6)	39,502/1,164	10,327,010 (49.5)	59 (27–222)	12 (4.8)	7
Being hit by moving objects	84,750 (18.3)	1393 (9.3)	26,725/433	1,904,359 (9.1)	45 (25–130)	51 (20.4)	60
Falls, trips, and slips	74,588 (16.1)	2627 (17.6)	32,543/830	3,758,039 (18.0)	54 (27–165)	15 (6.0)	20
Hitting objects with a part of the body	59,981 (12.9)	433 (2.9)	19,406/385	565,074 (2.7)	35 (22–74)	* (*)	3
Vehicle incidents	18,187 (3.9)	1055 (7.1)	46,553/1,004	1,424,469 (6.8)	73 (30–325)	122 (48.8)	671
Mental stress	14,415 (3.1)	1691 (11.3)	73,111/3,114	2,531,989 (12.1)	131 (51–355)	24 (9.6)	166
Chemicals and other substances	12,636 (2.7)	142 (0.9)	29,763/298	182,082 (0.9)	54 (24–231)	8 (3.2)	63
Heat, electricity, and other environment	12,473 (2.7)	113 (0.8)	20,329/363	116,152 (0.6)	30 (20–66)	14 (5.6)	112
Sound and pressure	6189 (1.3)	194 (1.3)	32,168/25,589	12,195 (0.1)	46 (27–157)	0 (0.0)	0
Biological factors	1983 (0.4)	31 (0.2)	15,302/366	35,588 (0.2)	36 (21–94)	* (*)	50
Other	113 (0.02)	3 (0.02)	23,734/329	4044 (0.02)	62 (20–331)	* (*)	885

*: Counts ≤5 were suppressed for privacy protection; Discrepancies may occur between sums of the component items and totals due to missing values.

**Table 5:** Summary of claims, percentages, compensation costs, and time-loss days by new worker, business size, first language, birth place, and season in South Australia, 2000–2014.

Category	Claims: n (%)	Total Cost (Million): n (%)	Median Cost for Serious/Non-Serious Claims	Total Time-Loss Days: n (%)	Time-Loss Days for Serious Claims: Median (25–75th Percentile)	Death: n (%)	Case Fatality Rate (per 100,000)
New worker *							
Yes	72,382 (15.6)	2735 (18.3)	36,861/455	4,201,254 (20.1)	64 (27–210)	50 (20.0)	69
No	391,757 (84.4)	12,199 (81.7)	37,530/744	16,659,747 (79.8)	58 (27–195)	200 (80.0)	51
Business size ^#^							
Large	238,715 (51.9)	6594 (44.7)	38,433/886	7,425,747 (35.6)	50 (25–142)	54 (21.6)	23
Medium	145,213 (31.6)	4561 (30.9)	36,572/506	7,101,593 (34.0)	65 (29–291)	84 (33.6)	58
Small	76,391 (16.6)	3583 (24.3)	35,233/563	6,142,663 (29.4)	73 (31–360)	107 (42.8)	140
First language							
English	451,802 (97.3)	14,241 (95.4)	36,758/668	19,851,622 (95.2)	58 (27–203)	247 (98.8)	55
Other	12,337 (2.7)	693 (4.6)	77,560/816	1,009,379 (4.8)	99 (33–528)	3 (1.2)	24
Born in Australia							
Yes	342,429 (73.4)	10,342 (69.3)	35,427/628	14,715,462 (70.5)	57 (27–200)	191 (76.4)	56
No	121,710 (26.2)	4592 (30.7)	43,180/828	6,145,539 (29.5)	62 (28–235)	59 (23.6)	48

*: New worker: the duration between injury date and start date with employer is less than 1 year or a worker is less or equal to 18 years old;.^#^: Employer size: the employer size is grouped by the number of employees as follows: small (1–20), medium (21–200), and large (≥2 01). Discrepancies may occur between sums of the component items and totals due to missing values.

## Supplementary Materials

The following are available online at https://www.mdpi.com/1660-4601/17/6/2015/s1, Table S1: Summary of claims, percentages, compensation costs, and time–loss days by age and gender, South Australia, 2000–2014; Table S2: Association between age and injury severity; Table S3: The number of claims, percentage, compensation payment, time–loss days, and deaths by birth place of an injured worker, South Australia, 2000–2014; Figure S1: Trends of serious workers’ compensation claim statistics in South Australia, 2000–2014: (A) age–standardised claim rates, (B) median compensation payments, (C) interquartile range of working days lost, and (D) the number of work–related deaths; Figure S2: Trends of non–serious workers’ compensation claim statistics in South Australia, 2000–2014: (A) age–standardised claim rates, (B) median compensation payments, (C) interquartile range of working days lost, and (D) the number of work–related deaths; Figure S3: Age-specific claim rates (per 100 employees) by industry, gender, and age group in South Australia, 2000–2014; Figure S4: Median compensation expenditure (AU$) for non–serious claims by industry, gender, and age group in South Australia, 2000–2014; Figure S5: Median compensation expenditure (AU$) for serious claims by industry, gender, and age group in South Australia, 2000–2014; Figure S6: Working days lost (interquartile range) due to serious claims by industry, gender, and age group in South Australia, 2000–2014; Figure S7: Age-specific claim rates (per 100 employees) by occupation, gender, and age group in South Australia, 2000–2014; Figure S8: Median compensation expenditure (AU$) for non–serious claims by occupation, gender, and age group in South Australia, 2000–2014; Figure S9: Median compensation expenditure (AU$) for serious claims by occupation, gender, and age group in South Australia, 2000–2014; Figure S10: Working days lost (interquartile range) due to serious claims by occupation, gender, and age group in South Australia, 2000–2014; Figure S11: Median compensation expenditure (AU$) for non–serious claims by nature of injury, gender, and age group in South Australia, 2000–2014; Figure S12: Median compensation expenditure (AU$) for serious claims by nature of injury, gender, and age group in South Australia, 2000–2014; Figure S13: Working days lost (interquartile range) due to serious claims by nature of injury, gender, and age group in South Australia, 2000–2014; Figure S14: Median compensation expenditure (AU$) for non–serious claims by mechanism of injury, gender, and age group in South Australia, 2000–2014; Figure S15: Median compensation expenditure (AU$) for serious claims by mechanism of injury, gender, and age group in South Australia, 2000–2014; Figure S16: Working days lost (interquartile range) due to serious claims by mechanism of injury, gender, and age group in South Australia, 2000–2014.
