# Supplementary material for: Characterising the Burden of Work-Related Injuries in South Australia: A 15-Year Data Analysis
Source: Int J Environ Res Public Health. 2020 Mar 18;17(6):2015. doi: 10.3390/ijerph17062015 (PMC7142853; doi:10.3390/ijerph17062015)
Supplement: Supplementary file 1 [file ijerph-17-02015-s001.pdf]

Supplementary tables and figures

Table S1: Summary of claims, percentages, compensation costs, and time-loss days by age and gender, South Australia, 2000-2014

				**Male**				
Age	Claims: n (%)	Claim rate (per 100)	Total cost (million): n (%)	Median cost for serious/non-serious claims	Total time-loss days: n (%)	Time-loss days for serious claims:: median (25-75th percentile)	Death: n (%)	Death rate (per 100,000)
15-24	58,015 (18.3)	5.9	639 (6.6)	18,800/424	981,504 (7.7)	40 (23-102)	20 (8.6)	2.0
25-34	76,699 (24.2)	6.4	1,920 (19.8)	32,525/558	2,644,808 (20.7)	51 (26-184)	35 (15.0)	2.9
35-44	79,269 (25.0)	6.7	2,960 (30.5)	41,775/695	3,792,565 (29.7)	55 (27-221)	64 (27.5)	5.4
45-54	65,405 (20.7)	5.9	2,810 (28.9)	44,354/892	3,602,206 (28.2)	60 (28-240)	62 (26.6)	5.6
55-64	34,231 (10.8)	5.3	1,310 (13.5)	44,354/1,149	1,673,755 (13.1)	63 (29-216)	45 (19.3)	6.9
65+	2,894 (0.9)	2.5	72 (0.7)	26,414/2,417	64,415 (0.5)	54 (28-126)	7 (3.0)	6.1
Sub-total	316,513 (100.0)	6.0	9,711 (100.0)	36,460/624	12,759,253 (100.0)	54 (26-195)	233 (100.0)	4.4
				**Female**				
Age	Claims: n (%)	Claim rate (per 100)	Total cost (million): n (%)	Median cost for serious/non-serious claims	Total time-loss days: n (%)	Time-loss days for serious claims:: median (25-75th percentile)	Death: n (%)	Death rate (per 100,000)
15-24	20,650 (14.0)	2.1	238 (4.6)	18,607/504	415,261 (5.1)	45 (24-109)	* (*)	0.2
25-34	26,214 (17.8)	2.6	718 (13.7)	34,339/718	1,094,756 (13.5)	60 (28-194)	* (*)	0.1
35-44	36,536 (24.8)	3.3	1,610 (30.8)	45,533/859	2,418,949 (29.9)	73 (30-278)	6 (35.3)	0.5
45-54	44,210 (30.0)	3.8	1,980 (37.9)	45,016/1,026	3,031,322 (37.4)	71 (31-266)	* (*)	0.4
55-64	19,059 (12.9)	3.2	660 (12.6)	40,172/1,050	1,111,226 (13.7)	68 (30-212)	* (*)	0.3

65+	930 (0.6)	1.4	16.7 (0.3)	25,456/1,043	28,412 (0.4)	59 (30-122)	* (*)	1.5
Sub-total	147,599 (100.0)	3.0	5,223 (100.0)	38,937/810	8,099,925 (100.0)	66 (29-231)	17 (100.0)	0.3

Total

Age	Claims: n (%)	Claim rate (per 100)	Total cost (million): n (%)	Median cost for serious/non-serious claims	Total time-loss days: n (%)	Time-loss days for serious claims:: median (25-75th percentile)	Death: n (%)	Death rate (per 100,000)
15-24	78,674 (17.0)	4.0	878 (5.9)	18,778/441	1,398,310 (6.7)	42 (24-105)	22 (8.8)	1.1
25-34	102,919 (22.2)	4.7	2,640 (17.7)	33,083/583	3,739,570 (17.9)	53 (26-187)	36 (14.4)	1.6
35-44	115,807 (25.0)	5.0	4,570(30.6)	43,050/738	6,211,727 (29.8)	61 (28-247)	70 (28.0)	3.0
45-54	109,623 (23.6)	4.8	4,790 (32.1)	44,692/943	6,633,586 (31.8)	65 (29-254)	67 (26.8)	3.0
55-64	53,292 (11.5)	4.3	1,970 (13.2)	42,590/1,112	2,784,981 (13.4)	65 (30-215)	47 (18.8)	3.8
65+	3,824 (0.8)	2.1	89 (0.6)	26,320/1,731	92,827 (0.4)	56 (29-125)	8 (3.2)	4.4
Total	464,139 (100.0)	4.6	14,937 (100.0)	37,428/671	20,861,001 (100.0)	58 (27-210)	250 (100.0)	2.5

Discrepancies may occur between sums of the component items and totals due to missing values.

*: Counts ≤5 were suppressed for privacy protection.

Table S2: Association between age and injury severity

Age group	Non-serious claims	Serious claims	% of serious claims	OR (95%CI)*
15-24	68,864	9,810	12.5	Reference
25-34	84,974	17,945	17.4	1.26 (1.22-1.29)
35-44	90,680	25,127	21.7	1.48 (1.44-1.52)
45-54	83,248	26,375	24.1	1.55 (1.50-1.59)
55-64	39,956	13,336	25.0	1.63 (1.58-1.69)
65+	2,988	836	21.2	1.42 (1.31-1.55)

* Industry, occupation, gender and nature of injury have been adjusted for serious claims.

Table S3: The number of claims, percentage, compensation payment, time-loss days, and deaths by birth place of an injured worker, South Australia, 2000-2014

Region	Claims: n (%)	Total cost (million): n (%)	Median cost for serious/non-serious claims	Total time-loss days: n (%)	Time-loss days for serious claims:: median (25-75th percentile)	Death: n (%)	Case fatality rate (per 100,000)
Australia	342,429 (73.8)	10,300 (69.1)	35,427/628	14,700,000 (70.3)	57 (27 - 200)	191 (76.4)	56
Africa	2,811 (0.6)	108 (0.7)	38,584/723	166,487 (0.8)	61 (28 - 247)	0 (0.0)	0
Central-South Asia	3,377 (0.7)	86 (0.6)	31,348/648	141,373 (0.7)	57 (26 - 197)	* (*)	30
North-East Asia	1,352 (0.3)	50 (0.3)	30,877/717	68,732 (0.3)	58 (30 - 200)	* (*)	148
South-East Asia	5,993 (1.3)	271 (1.8)	58,664/808	372,120 (1.8)	75 (32 - 340)	0 (0.0)	0
Middle East	1,040 (0.2)	113 (0.8)	195,852/891	171,894 (0.8)	343 (58 - 727)	0 (0.0)	0
Europe	53,767 (11.6)	2,410 (16.2)	49,306/857	3,280,994(15.7)	67 (29 - 298)	26 (10.4)	48
North America	1,163 (0.3)	47 (0.3)	46,435/703	64,133 (0.3)	75 (30 - 218)	0 (0.0)	0
South/Latin America	1,324 (0.3)	59 (0.4)	50,103/836	88,762 (0.4)	81 (34 - 108)	0 (0.0)	0
Pacific Island Countries	6,416 (1.4)	216 (1.4)	35,497/616	306,200 (1.5)	59 (27 - 211)	7 (2.8)	109
Unknown	44,467 (9.6)	1,240 (8.3)	35,824/873	1,484,844	54 (27 - 155)	23 (9.2)	52
Total	464,139 (100.0)	14,934 (100.0)	37,428/671	20,861,003 (100.0)	58 (27 - 210)	250 (100.0)	54

Discrepancies may occur between sums of the component items and totals due to missing values.

*: Counts ≤5 were suppressed for privacy protection.

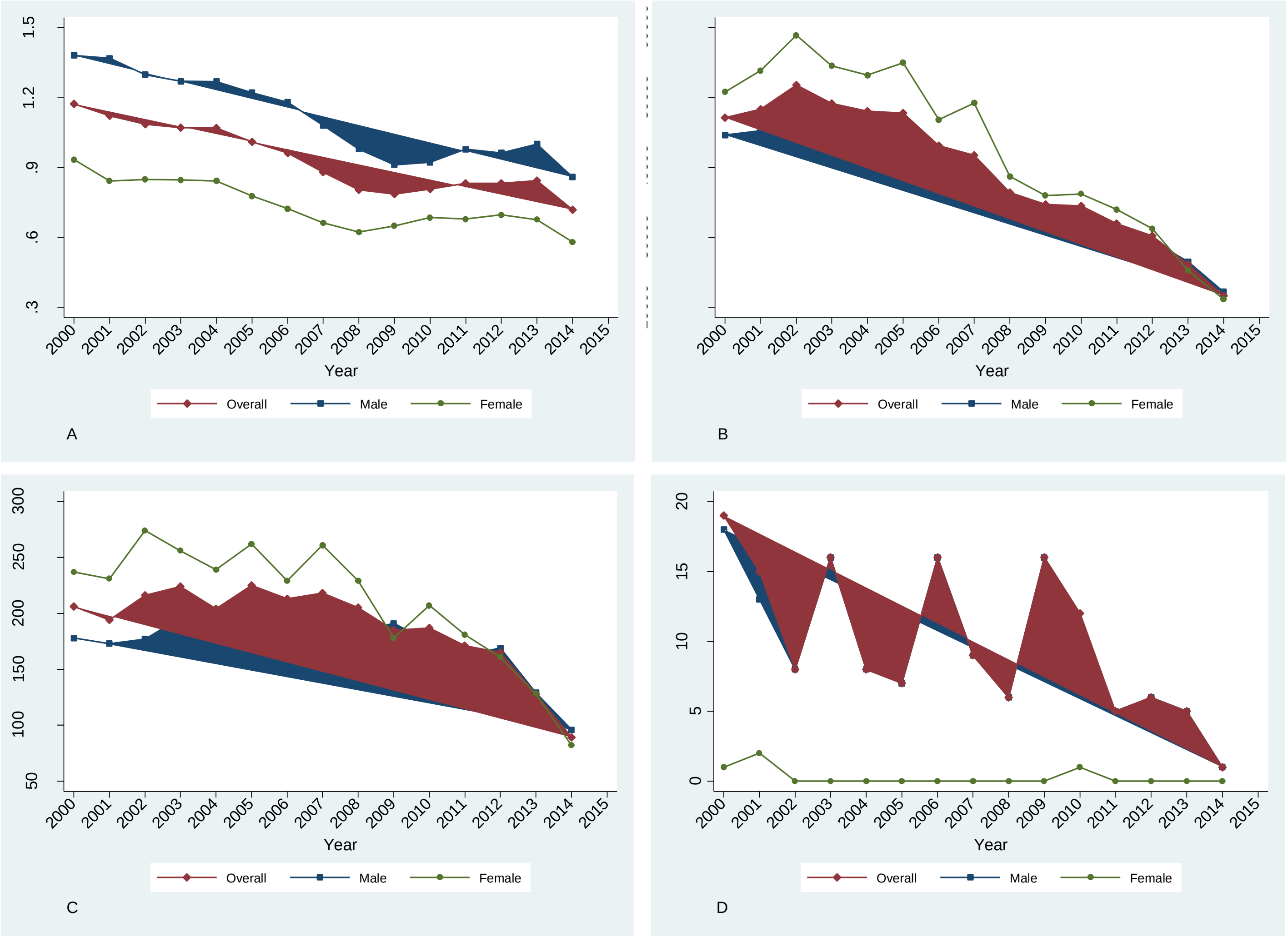

Figure S1: Trends of **serious** workers' compensation claim statistics in South Australia, 2000-2014: (A) age-standardised claim rates, (B) median compensation payments, (C) interquartile range of working days lost, and (D) the number of work-related deaths.

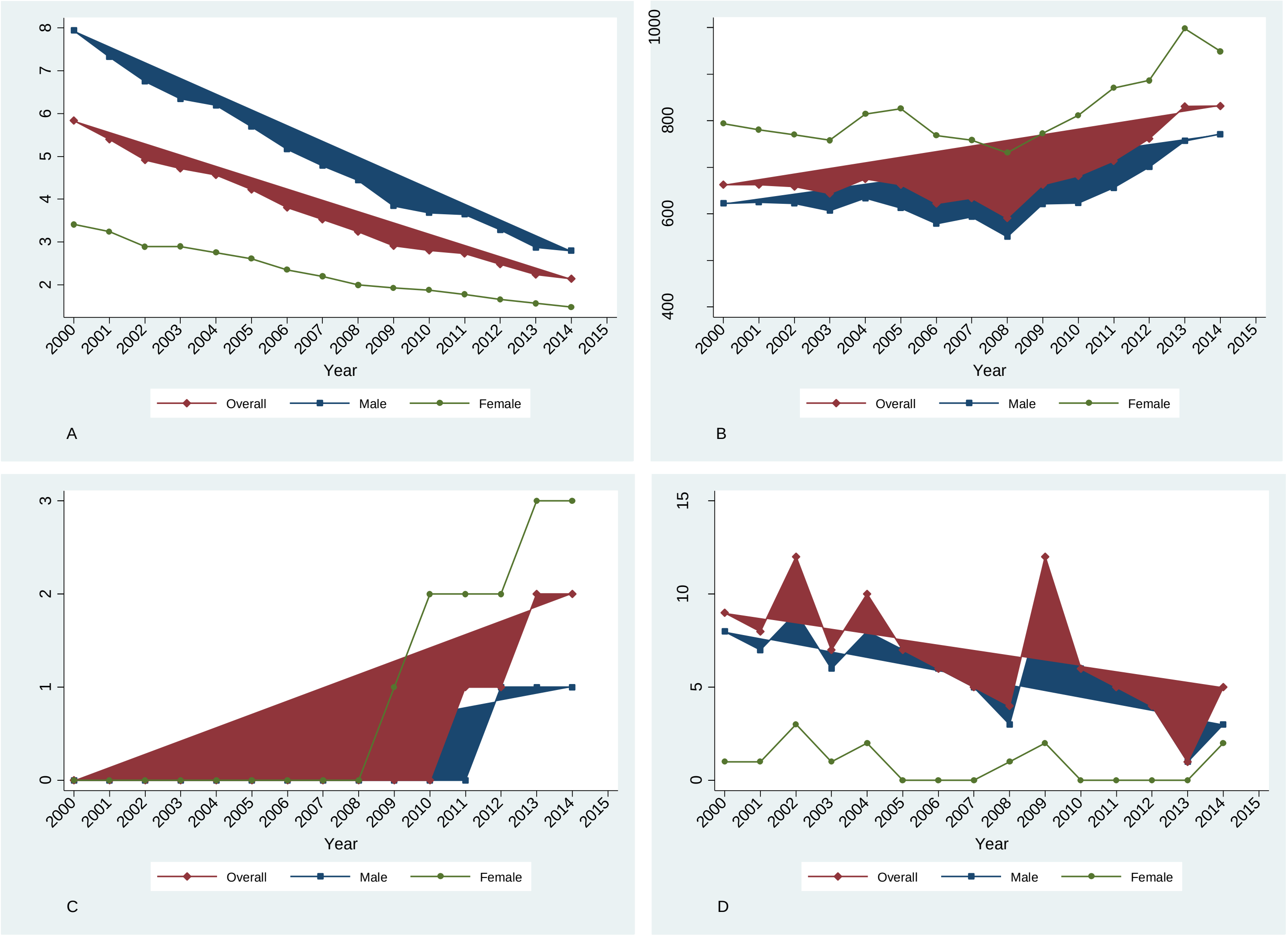

Figure S2: Trends of **non-serious** workers' compensation claim statistics in South Australia, 2000-2014: (A) age-standardised claim rates, (B) median compensation payments, (C) interquartile range of working days lost, and (D) the number of work-related deaths.

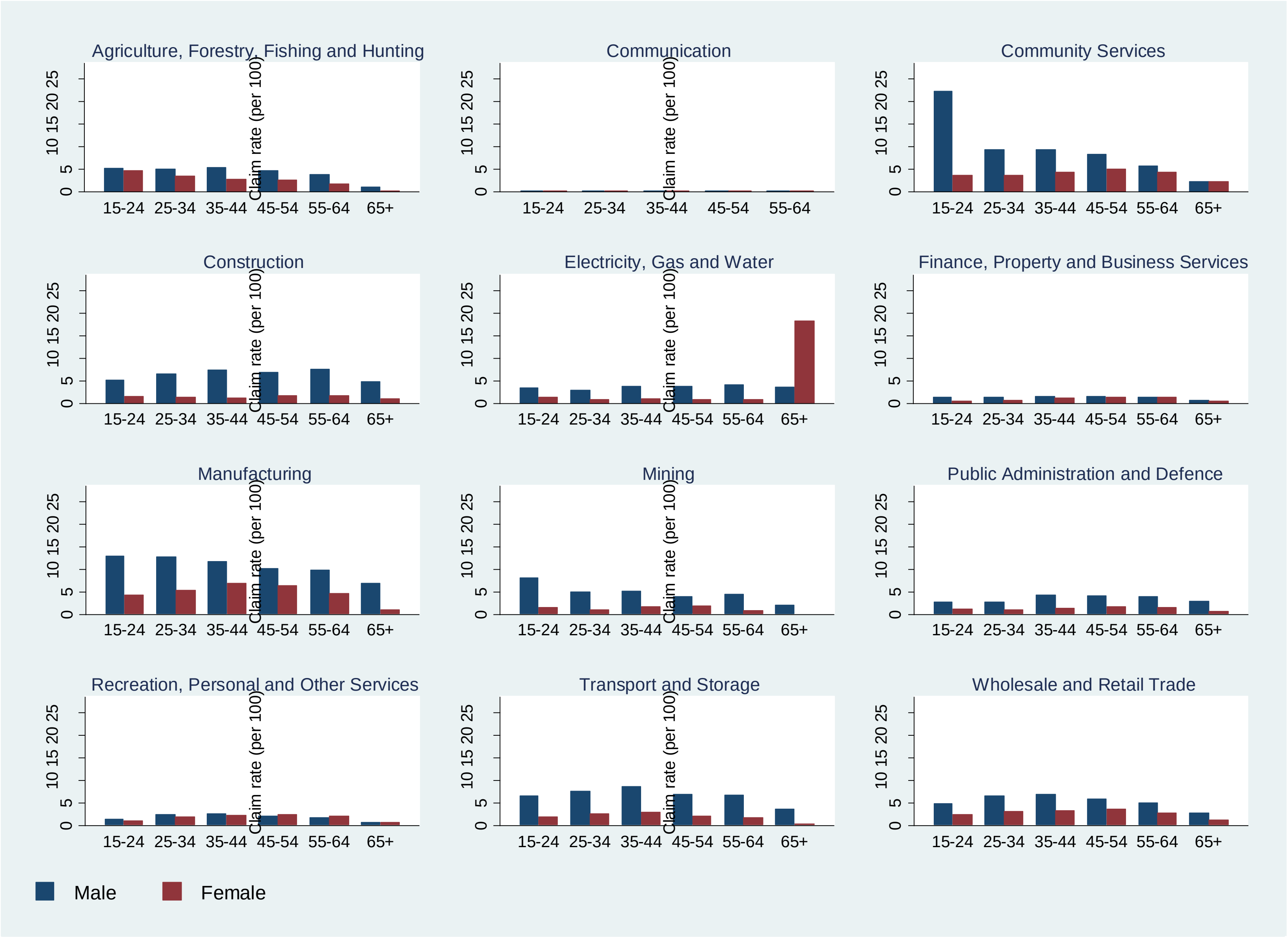

Figure S3: Age-specific claim rates (per 100 employees) **by industry**, gender, and age group in South Australia, 2000-2014

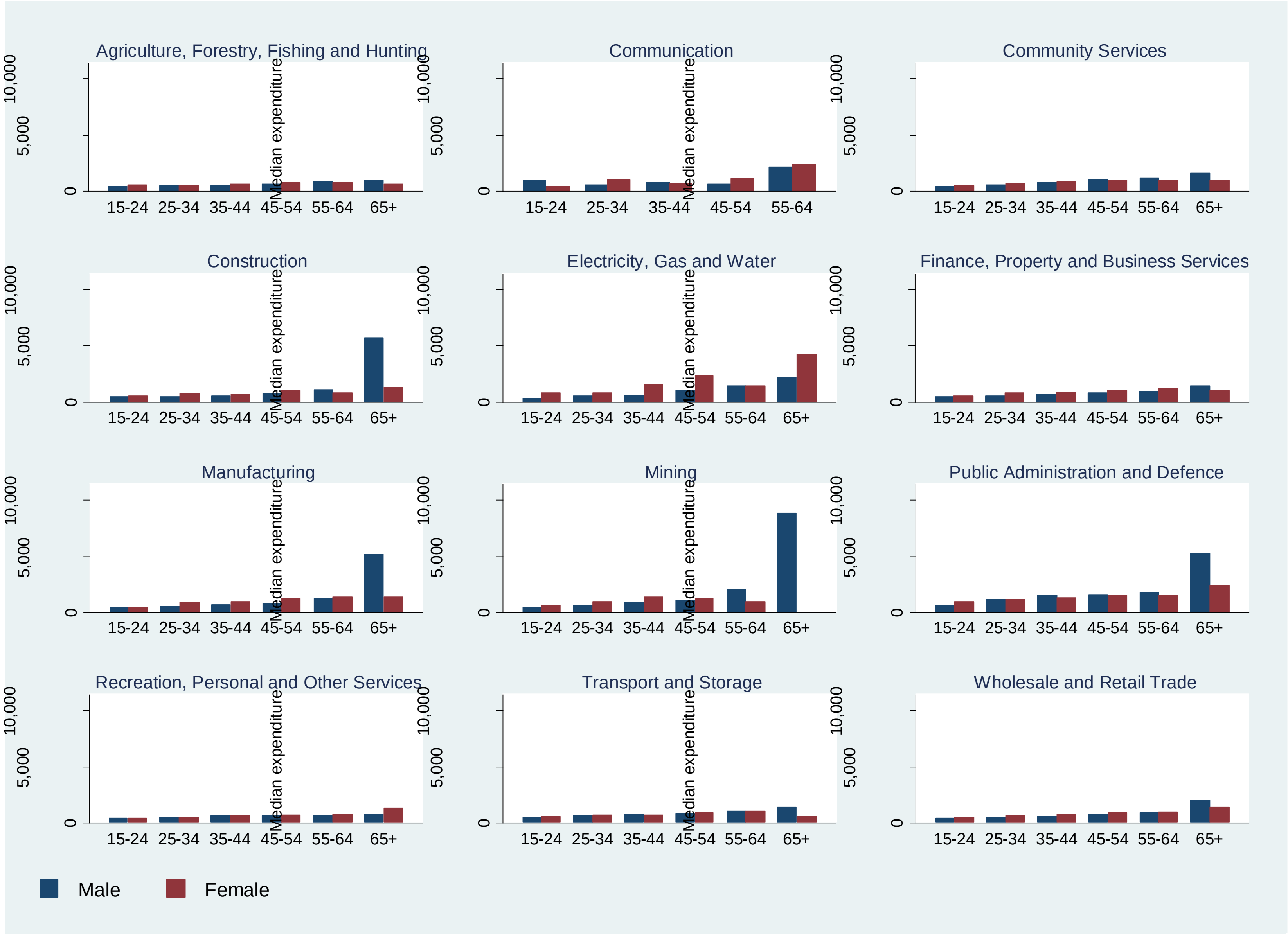

Figure S4: Median compensation expenditure (AU$) for **non-serious claims by industry**, gender, and age group in South Australia, 2000-2014

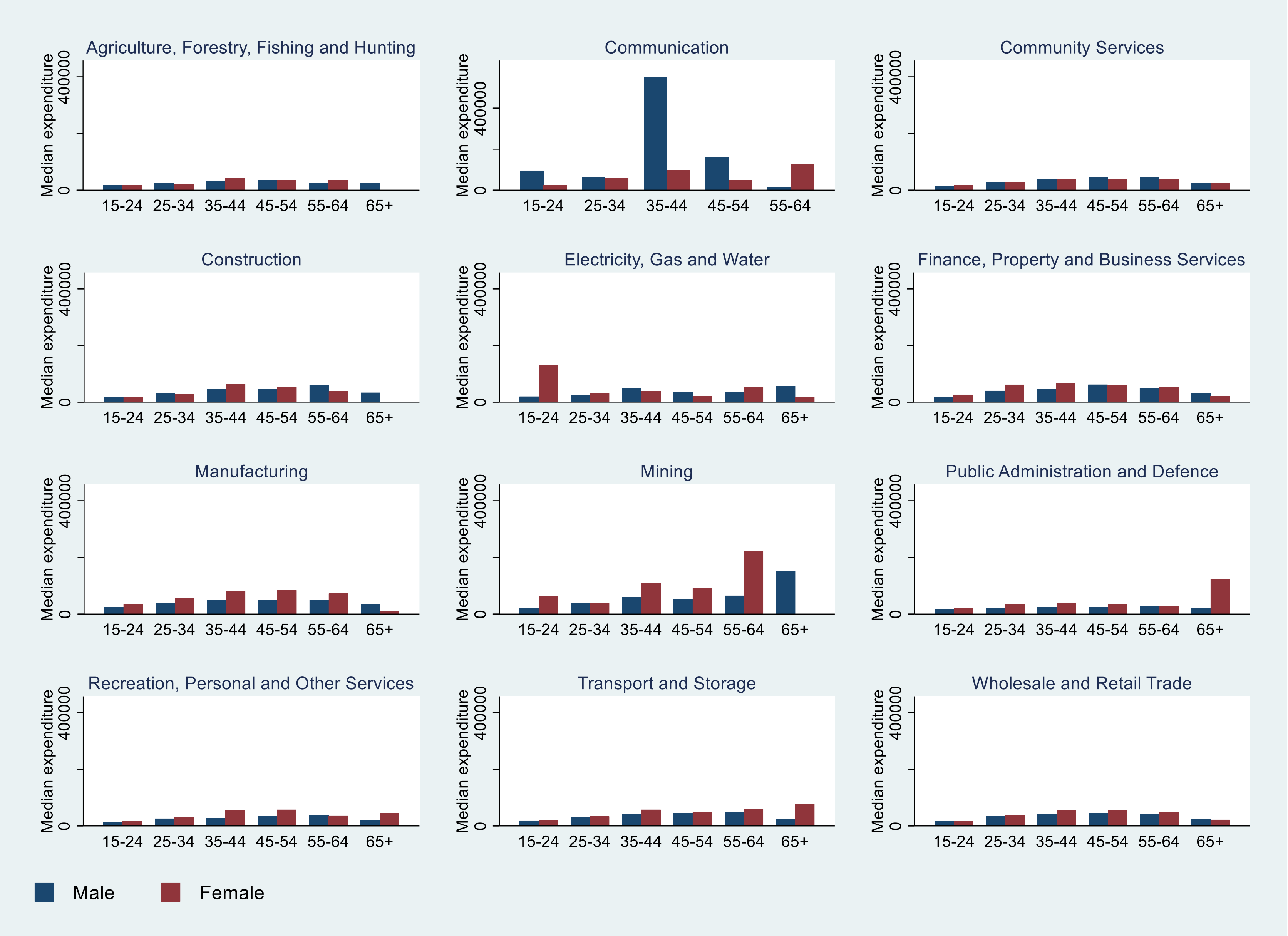

Figure S5: Median compensation expenditure (AU$) for **serious claims by industry**, gender, and age group in South Australia, 2000-2014

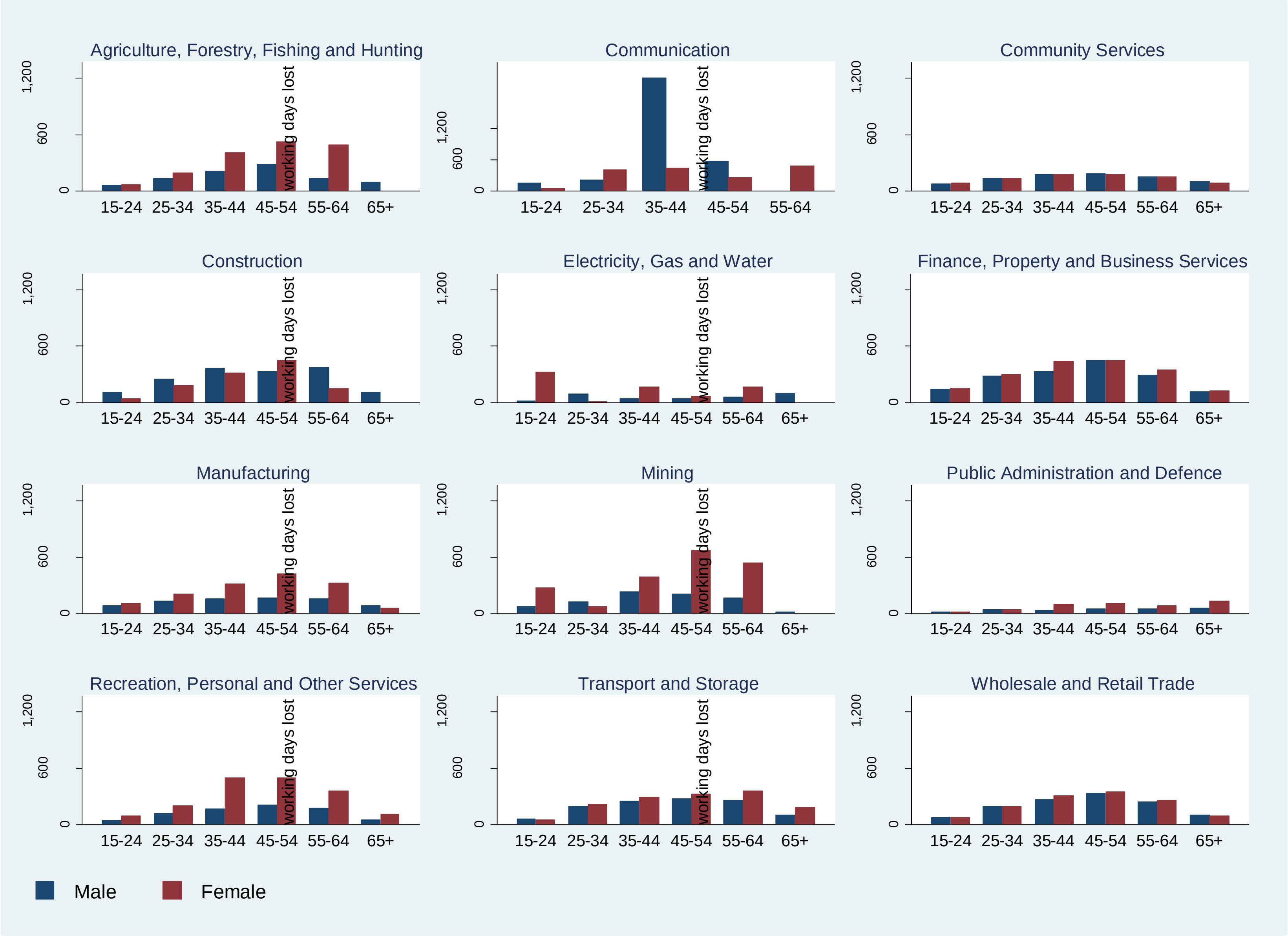

Figure S6: Working days lost (interquartile range) due to **serous claims by industry**, gender, and age group in South Australia, 2000-2014

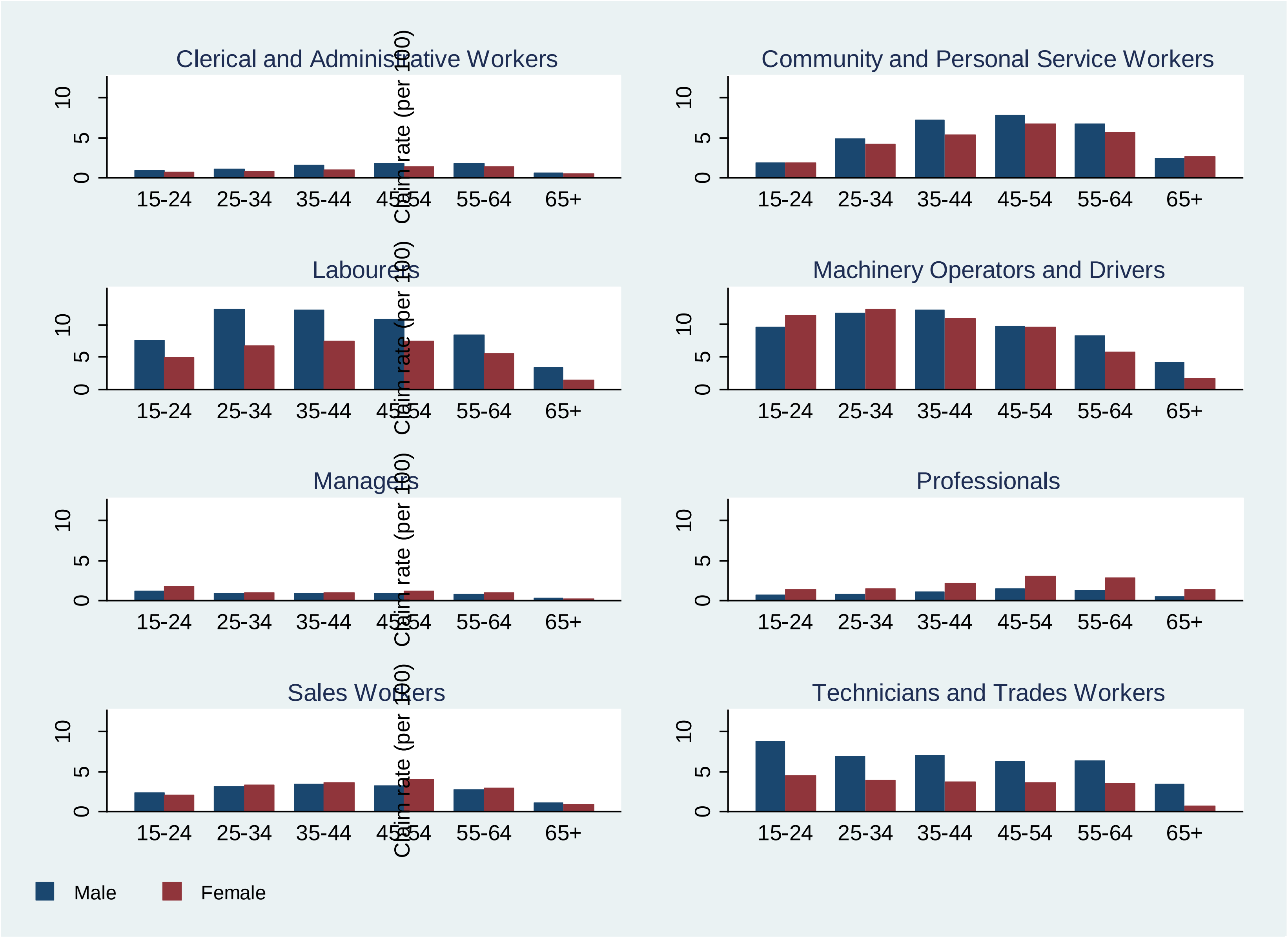

Figure S7: Age-specific claim rates (per 100 employees) **by occupation**, gender, and age group in South Australia, 2000-2014

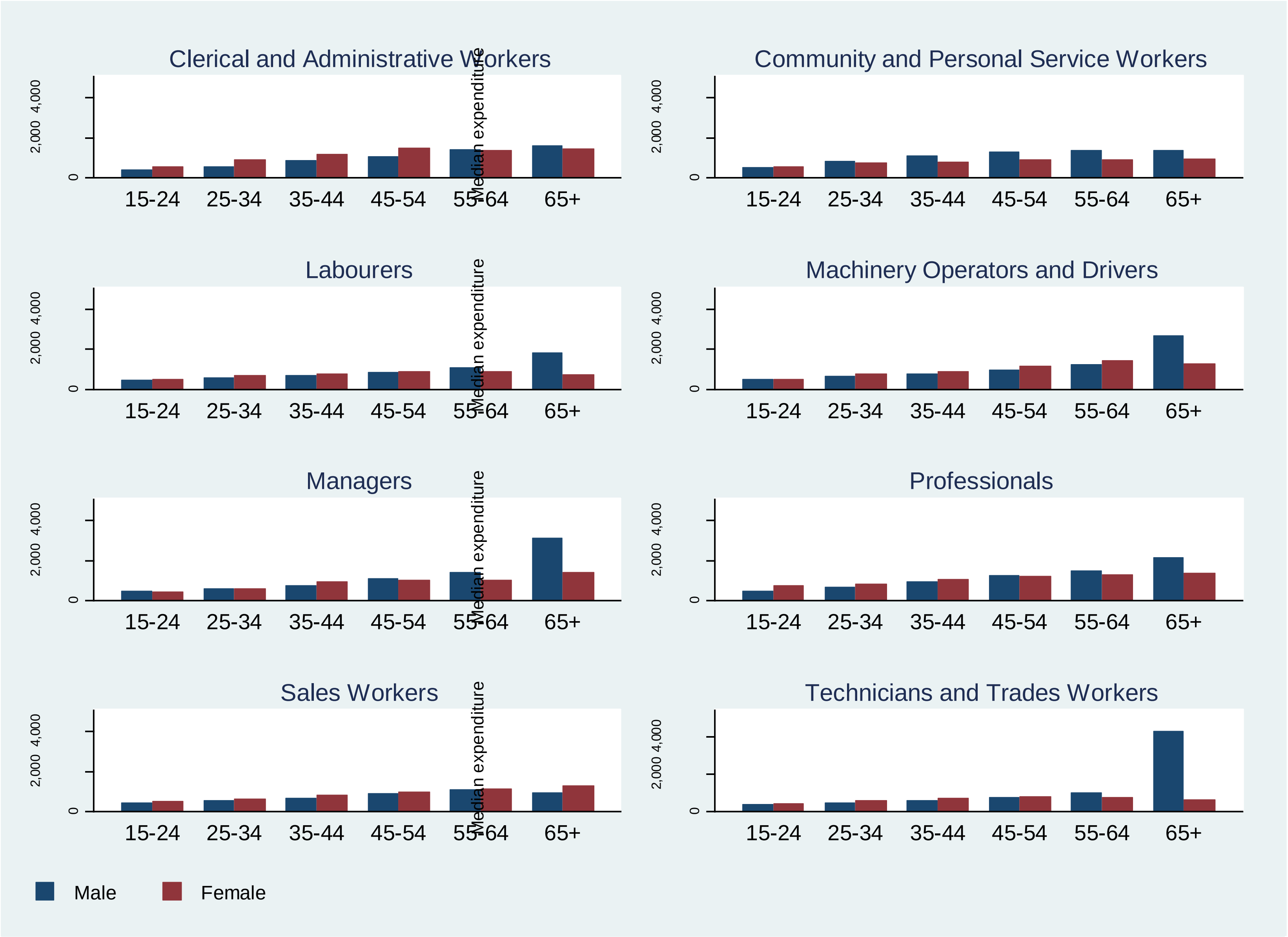

Figure S8: Median compensation expenditure (AU$) for **non-serious claims by occupation**, gender, and age group in South Australia, 2000-2014

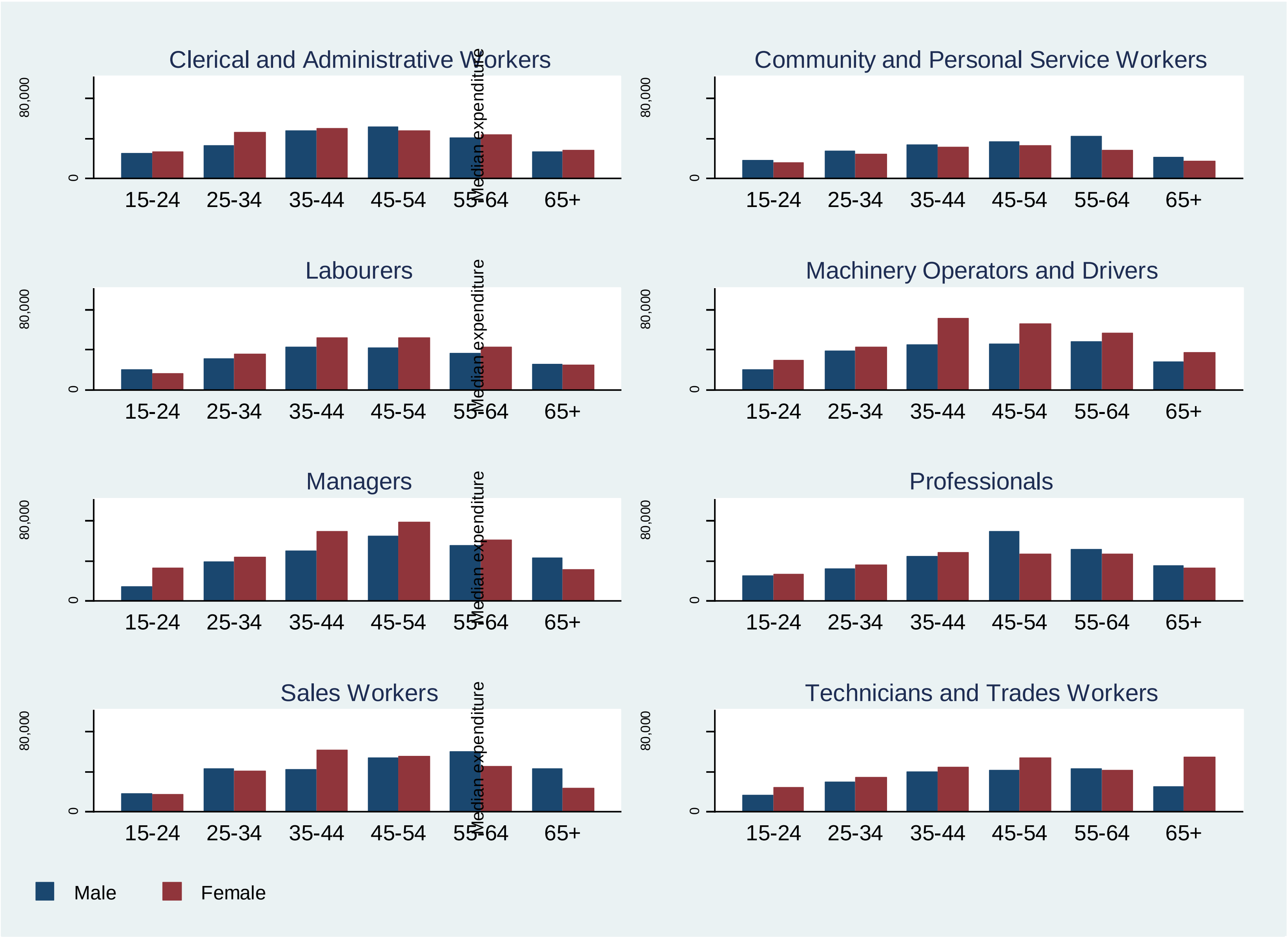

Figure S9: Median compensation expenditure (AU$) for **serious claims by occupation**, gender, and age group in South Australia, 2000-2014

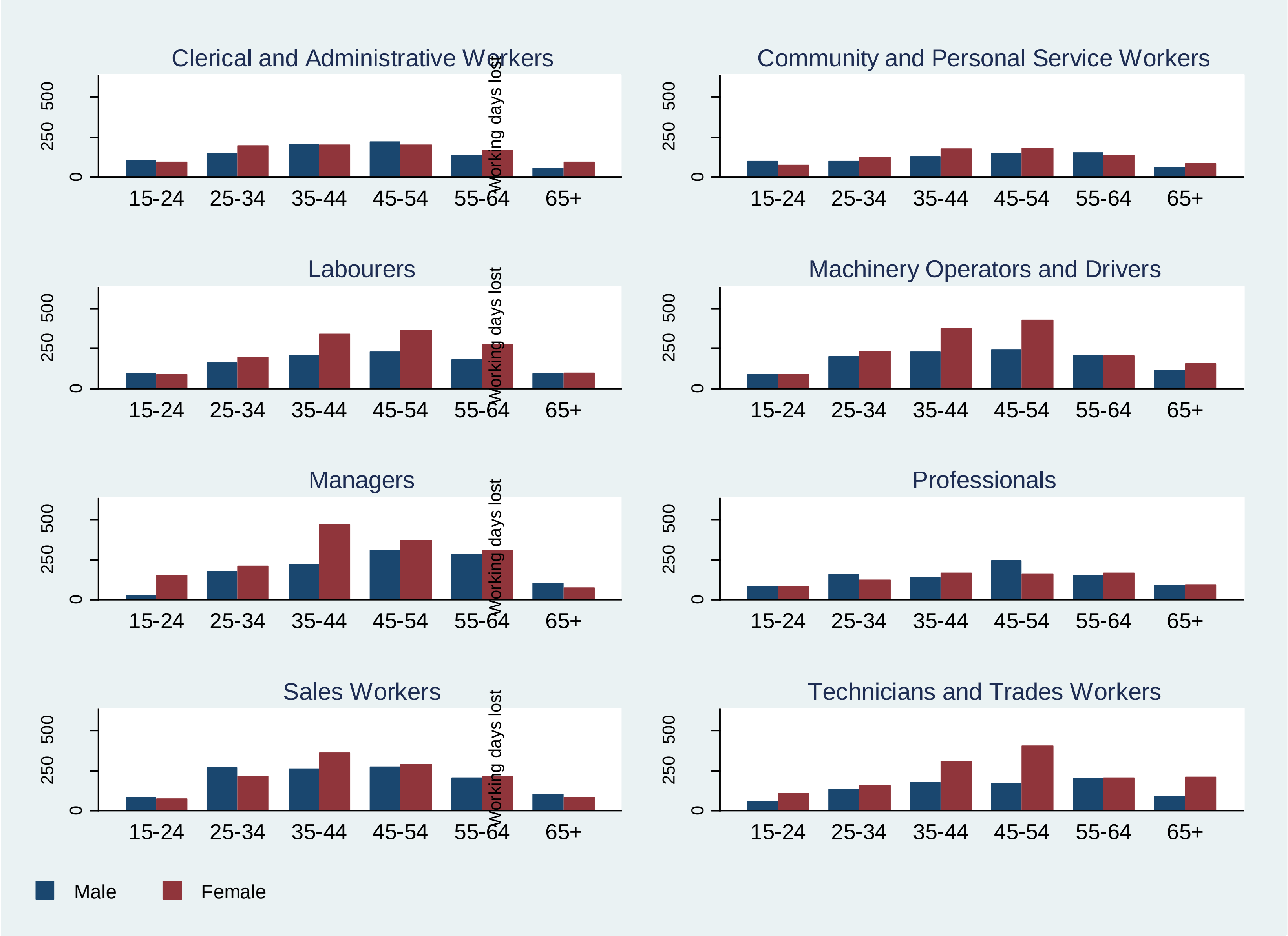

Figure S10: Working days lost (interquartile range) due to **serious claims by occupation**, gender, and age group in South Australia, 2000-2014

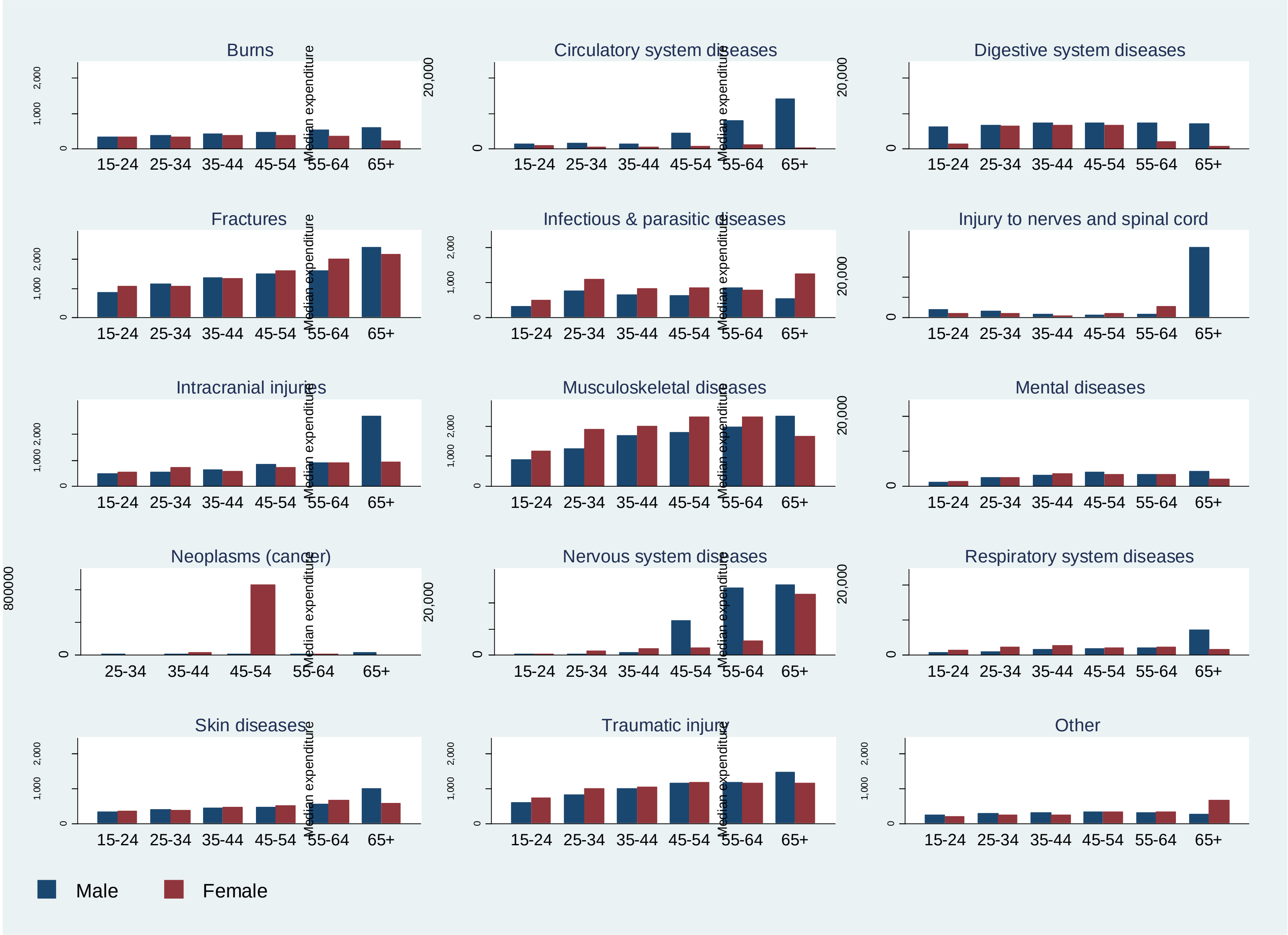

Figure S11: Median compensation expenditure (AU$) for **non-serious claims by nature of injury**, gender, and age group in South Australia, 2000-2014

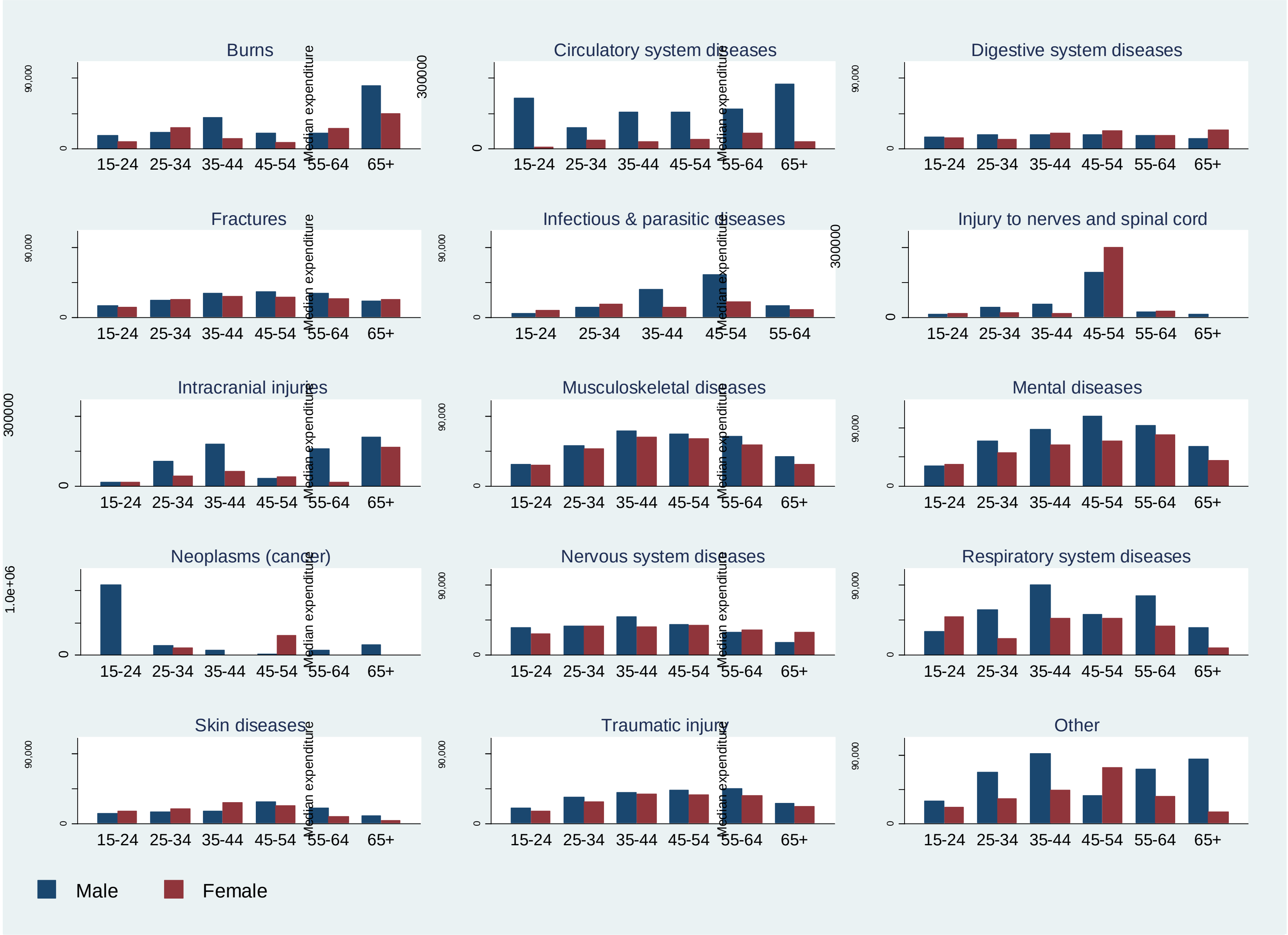

Figure S12: Median compensation expenditure (AU$) for **serious claims by nature of injury**, gender, and age group in South Australia, 2000-2014

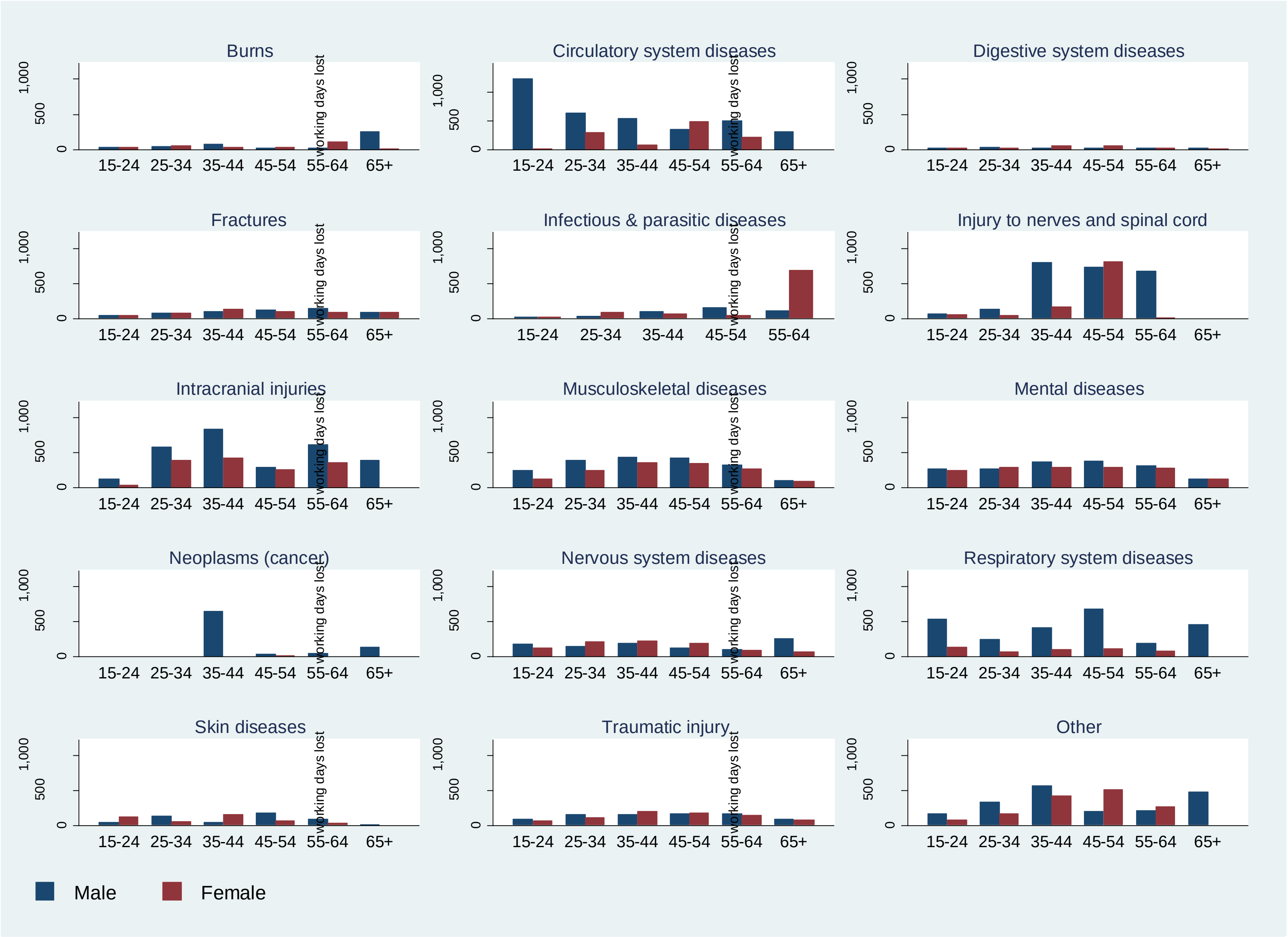

Figure S13: Working days lost (interquartile range) due to **serious claims by nature of injury**, gender, and age group in South Australia, 2000-2014

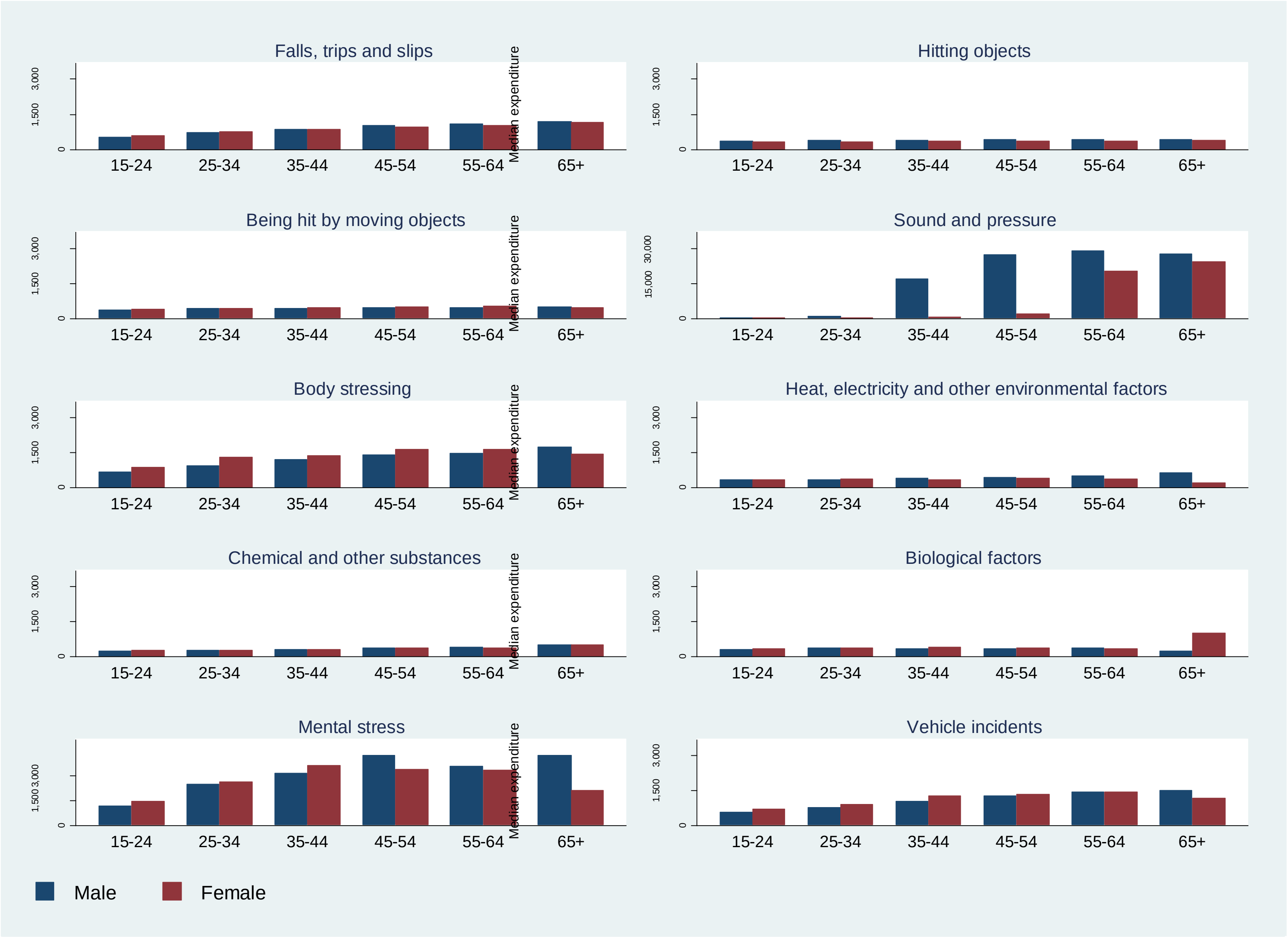

Figure S14: Median compensation expenditure (AU$) for **non-serious claims by mechanism of injury**, gender, and age group in South Australia, 2000-2014

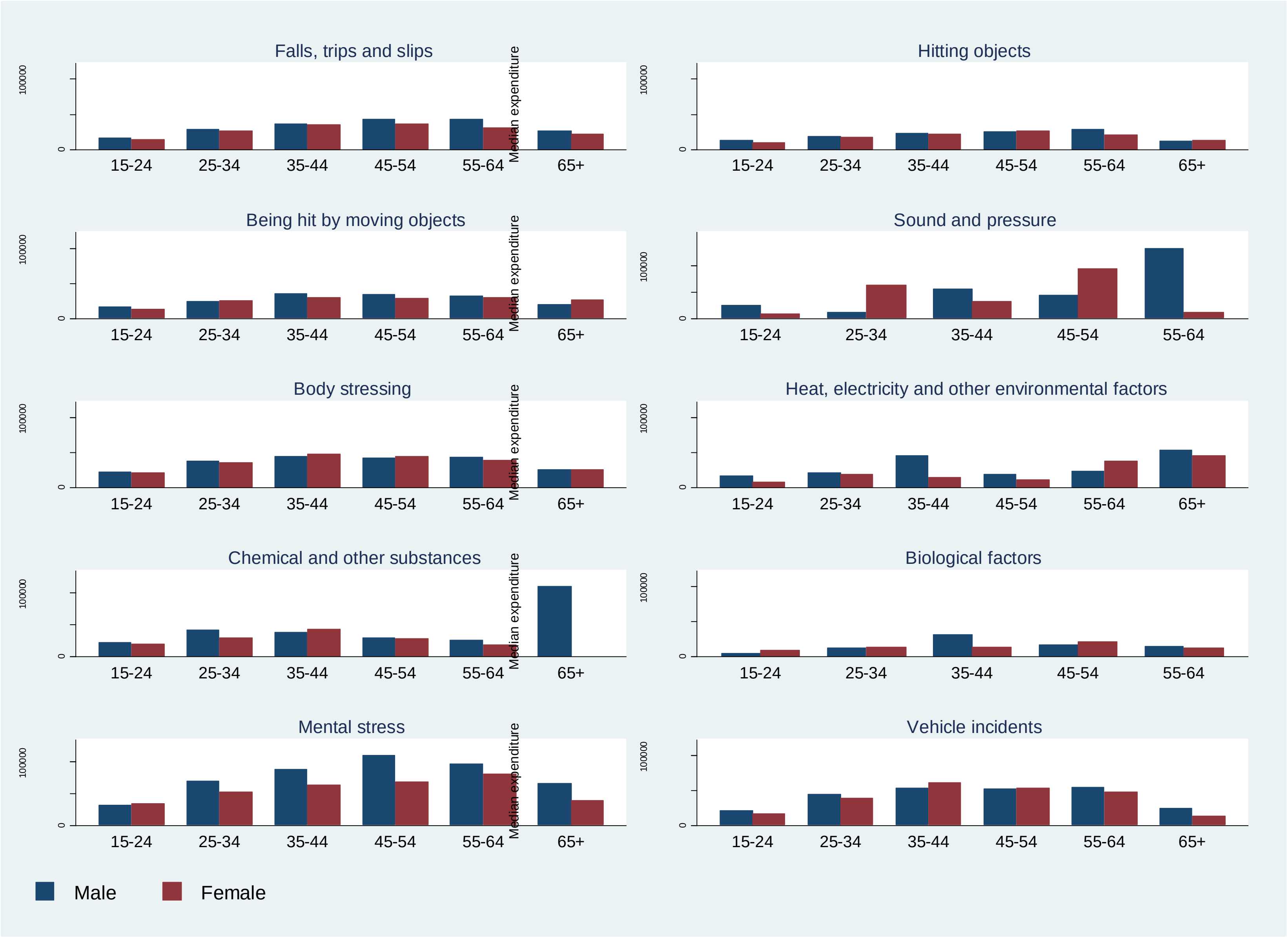

Figure S15: Median compensation expenditure (AU$) for **serious claims by mechanism of injury**, gender, and age group in South Australia, 2000-2014

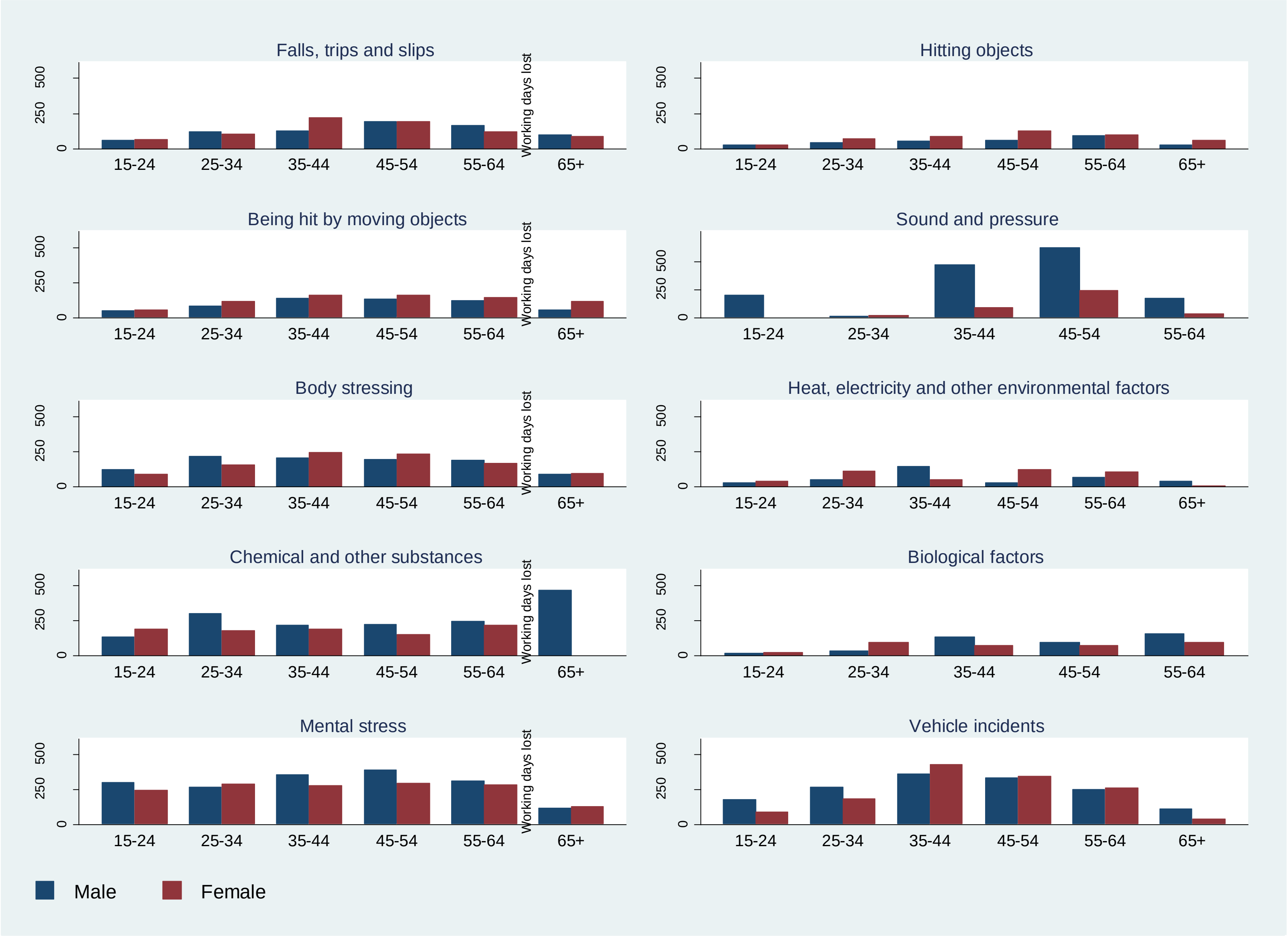

Figure S16: Working days lost (interquartile range) due to **serious claims by mechanism of injury**, gender, and age group in South Australia, 2000-2014
